# *Brucella abortus* modulates macrophage polarization and inflammatory response by targeting glutaminases through the NF-κB signaling pathway

**DOI:** 10.3389/fimmu.2023.1180837

**Published:** 2023-05-31

**Authors:** Tianyi Zhao, Zedan Zhang, Yitao Li, Zhihua Sun, Liangbo Liu, Xingmei Deng, Jia Guo, Dexin Zhu, Shuzhu Cao, Yingjin Chai, Usevich Vera Nikolaevna, Suleimenov Maratbek, Zhen Wang, Hui Zhang

**Affiliations:** ^1^ State International Joint Research Center for Animal Health Breeding, College of Animal Science and Technology, Shihezi University, Shihezi, China; ^2^ College of Veterinary, Ural State Agricultural University, Yekaterinburg, Russia; ^3^ College of Veterinary, National Agricultural University of Kazakhstan, Nur Sultan, Kazakhstan

**Keywords:** *Brucella abortus*, cell polarization, NF-κB, ChIP-seq, glutaminase

## Abstract

**Objectives:**

The mechanism of Brucella infection regulating macrophage phenotype has not been completely elucidated until now. This study aimed to determine the mechanism of *Brucella abortus* in the modulation of macrophage phenotype using RAW264.7 cells as a model.

**Materials and methods:**

RT-qPCR, ELISA and flow cytometry were used to detect the inflammatory factor production and phenotype conversion associated with M1/M2 polarization of macrophages by *Brucella abortus* infection. Western blot and immunofluorescence were used to analyze the role of nuclear factor kappa B (NF-κB) signaling pathway in regulation of *Brucella abortus*-induced macrophage polarization. Chromatin immunoprecipitation sequencing (Chip‐seq), bioinformatics analysis and luciferase reporter assay were used to screen and validate NF-κB target genes associated with macrophage polarization and further verify its function.

**Results:**

The results demonstrate that *B. abortus* induces a macrophage phenotypic switch and inflammatory response in a time-dependent manner. *With the increase of infection time*, *B. abortus* infection-induced M1-type increased first, peaked at 12 h, and then decreased, whereas the M2-type decreased first, trough at 12 h, and then increased. The trend of intracellular survival of *B. abortus* was consistent with that of M2 type. When NF-κB was inhibited, M1-type polarization was inhibited and M2-type was promoted, and the intracellular survival of *B. abortus* increased significantly. Chip‐seq and luciferase reporter assay results showed that NF-κB binds to the glutaminase gene (*Gls*). *Gls* expression was down-regulated when NF-κB was inhibited. Furthermore, when *Gls* was inhibited, M1-type polarization was inhibited and M2-type was promoted, the intracellular survival of *B. abortus* increased significantly. Our data further suggest that NF-κB and its key target gene *Gls* play an important role in controlling macrophage phenotypic transformation.

**Conclusions:**

Taken together, our study demonstrates that *B. abortus* infection can induce dynamic transformation of M1/M2 phenotype in macrophages. Highlighting NF-κB as a central pathway that regulates M1/M2 phenotypic transition. This is the first to elucidate the molecular mechanism of *B. abortus* regulation of macrophage phenotype switch and inflammatory response by regulating the key gene *Gls*, which is regulated by the transcription factor NF-κB.

## 1 Introduction

Brucellosis is a global zoonotic disease caused by gram-negative, intracellular bacteria of the genus *Brucella* (1). The disease also severely affects animal reproduction, resulting in huge economic losses for the livestock industries of developing countries (2). Macrophages are among the most important cells involved in pathogen recognition and clearance in the innate immune response and are also the main host cells of *B. abortus* (3). *Brucella* can invade and replicates within host macrophages. To survive and establishes infection in the hostile macrophage intracellular environment, *Brucella* adopted several strategies. After entering the intracellular, *Brucella* by surviving within a membranous compartment, the *Brucella*-containing vacuole, thus avoiding fusion with the lysosomal compartments leading to its degradation (4). *B. abortus* prolonging replication time by inhibiting apoptosis of infected macrophages, thereby promotes intracellular survival (1). In addition, *Brucella* establish a favorable intracellular environment for its intracellular survival by using autophagy mechanism (5). Recent studies have shown that *Brucella melitensis M5* can modulates the macrophages phenotypes and conducive to intracellular survival, but its specific molecular mechanism has not yet been fully clarified (6).

Macrophages play key roles in resolving inflammation, defending against pathogens, and maintaining the stability of the internal environment (7). Highly plastic macrophages can be induced to classified into classically activated (M1) and alternatively activated (M2) phenotypes, in response to different tissue microenvironment and/or pathogenic stimuli (8). Induced by lipopolysaccharide (LPS) and/or interferon-γ (IFN- γ), M1 macrophages can secrete inducible interleukin 1β (IL-1β), the cytokines tumor necrosis factor α (TNF-α) and nitric oxide synthase (NOS2; iNOS), which mainly exert pro-inflammatory and cytotoxic effects (9). Induced by interleukin 4 (IL-4), M2 macrophages secrete interleukin 10 (IL-10), transforming growth factor-β (TGF-β), and inducible arginase 1 (ARG1), which primarily exert anti-inflammatory and tissue remodeling processes (10).

The mechanism of regulating macrophages polarization phenotypes is complex, involving interactions among multiple signaling molecules and pathways (11). Signal transducers and activators of transcription (STAT) 1 and NF-κB mainly mediate the regulation of M1 macrophage polarization (12), whereas the regulation of M2 is mainly mediated by STAT6 (13). NF-κB plays an important role in regulating the expression of M1 pro-inflammatory genes (including *Tnf-a*, *Nos2*, and *Il1β*) (14), and phosphorylated NF-κB migrates to the nucleus and regulates polarization by inducing the expression of downstream target genes. In an LPS-induced lung injury model, downregulation of NF-κB expression inhibited the polarization of M1 macrophages, thus alleviating acute lung injury (15). Some drugs such as nicorandil are effective regulators of the NF-κB signaling pathway and also regulate the M1/M2 status (16).

Studies have shown that pathogen infection can cause macrophage polarization. The M1 pro-inflammatory phenotype of macrophages has been widely reported in infections of several pathogens such as *Enterococcus faecalis*, *Listeria monocytogenes*, *Salmonella* and *Chlamydia* (17–20). *Salmonella typhimurium* tends to stimulate macrophages to polarize to the M2 type (21). In *Mycobacterium tuberculosis*-infected macrophages, the M1 phenotype is upregulated in the early stage of infection, whereas the M2 phenotype is dominant in the middle and late stages (22). Studies have shown that *Brucella* infection can also cause macrophage polarization. Stimulator of interferon genes can promote the polarization of inflammatory M1-type phenotype by regulating metabolic reprogramming of *Brucella*-infected macrophages by hypoxia-inducible factor-1 alpha (3). In addition, *Brucella* can also inhibit macrophage polarization by LC3-dependent autophagy (5). Another study showed that M1-type polarization increased in the early stages of *Brucella* infection, whereas in the later stages of infection it showed an increase in M2-type polarization due to STAT6-mediated conversion of the M2 phenotype (6). Although some reports have investigated some mechanisms of Brucella-induced macrophage polarization and inflammation, there are still many mechanisms that have not been elucidated and still need further study. NF-κB is a key transcriptional regulator that regulates the polarization of macrophages (23). Research have shown that some pathogen such as *Staphylococcus aureus* can transform macrophages from the M1 to M2 type by inhibiting NF-κB activity (24). *Mycobacterium tuberculosis* can inhibit the activation of M1 macrophages by inhibiting phagosomal maturation and NF-κB activity (25).

These findings further emphasize the role of NF-κB in regulating cell polarization. As an important regulator, the mechanism by which NF-κB regulates cell polarization is likely to involve the transcriptional regulation of key genes involved in polarization. However, it is unclear whether *B. abortus* can also activate the NF-κB signaling pathway and thus mediate macrophage polarization *via* NF-κB. Thus, the aim of this study was to investigate the relationship between NF-κB and macrophage polarization in *B. abortus* during infection and to elucidate the mechanisms by which Brucella regulates macrophage phenotype.

## 2 Materials and methods

### 2.1 Bacterial strains and media

The virulent *Brucella abortus* strain 2308 (S2308) was obtained from the Center of Chinese Disease Prevention and Control (Beijing, China). *B. abortus* was cultured on tryptic soy agar (TSA) plates or in tryptic soy broth (TSB) medium (Difco, MI, USA) at 37°C in a 5% CO_2_ atmosphere. All analyses of live S2308 were conducted in a biosafety level-3 laboratory.

### 2.2 Cell culture and infection

The murine macrophage line RAW 264.7 (Cell Resource, Beijing, China) was cultured in Dulbecco’s modified Eagle medium (DMEM; Gibco Life Technologies) supplemented with 10% fetal bovine serum (FBS; Gibco) at 37°C, 5% CO_2_ (v/v). The macrophages were infected with *B. abortus* with a multiplicity of infection (MOI) of 100:1 and then incubated at 37°C in 5% CO_2_ for 60 min. Fresh DMEM medium (containing 50 μg/mL gentamicin) was added after washing with phosphate-buffered saline (PBS) twice. After incubation at 37°C for 45 min, the cells were washed again with PBS twice. Fresh DMEM (defined as time zero) was added and the cells were further incubated at 37°C. Cultures solution were aspirated and discarded at 0, 4, 8, 12, 24, and 48 hours after *B. abortus* infection. The cells were then washed three times with PBS and lysed using Triton with a 0.1% (v/v) concentration. Live bacterial lysates were diluted in a 10-fold gradient and incubated on TSA for counting to quantify bacterial survival.

### 2.3 Cell viability assay

Macrophages (1 × 10^5^ cells/well) were exposed to various concentrations of an NF-κB signaling pathway inhibitor BAY (BAY 11-7082, # HY-13453) or glutaminase (*Gls*) inhibitor BPTES (MedChemExpress, New Jersey, USA # HY-12683) in DMEM. The medium was replaced with fresh DMEM after 24 h culture. CCK-8 reagent (10 μL/well) (Beytotime, Jiangsu, China) was then added to the culture medium, followed by incubation for 4 h. The optical density at 450 nm was read on an automated microplate reader (Tecan Sunrise, CH).

### 2.4 Total RNA extraction and reverse transcription-quantitative polymerase chain reaction

Total cellular RNA from RAW264.7 was isolated using TRIzol reagent (Takara, Japan), and the first strand cDNA was synthesized by reverse transcription using a PrimeScript RT kit (Takara, Japan). SYBR Green Master Mix (Promega, Beijing, China) and a Bio-RAD IQ5 system were used to conduct qPCR in a 10-μL reaction system, and the related target genes in RAW264.7 cells were detected. All experiments were performed in triplicate. The 2^-ΔΔCt^ cycle threshold method was used to normalize the relative expression levels of mRNAs to those of the internal control GAPDH. The primer sequences are listed in **Supplementary Table 1A**.

### 2.5 Flow cytometry

*B. abortus* infection of macrophages for 12 and 48 hours as described in the “Cell Culture and Infection” section of the materials and methods. Aspirate and discard the old culture medium, add 1 mL digestive juice (trypsin) for 1 min and then add 1 mL of fresh DMEM, collect the cell suspension into a centrifuge tube, centrifuge at 200 ×g for 5 min, discard the supernatant and keep the cell precipitate. Macrophages precipitate were washed twice with 1X Binding Buffer to determine M1 and M2 macrophage marker CD86 and CD206. Each set of cell samples was further divided equally into two groups, one for labeling CD86 alone and the other for labeling CD206 alone. CD86 individually labeled group cells were first added to a 400 μL aliquot of a Cell Fixation Reagent (BD, USA), incubated at 4 °C for 15 min in the dark, then centrifuge at 150 ×g for 5 min and washed twice with 1 X Binding Buffer. Fluorescein Isothiocyanate-labeled anti-mouse CD86 (Thermo Fisher, USA) was added to the cells and incubated in the dark for 30 minutes. CD206 individually labeled group cells were added to 400 μL aliquots of Cell Fixation Reagent and incubated for 15 min at 4°C in the dark. Centrifuge at 150 ×g for 5 min, wash twice with 1x binding buffer, add 200 μL Cell Membrane-Breaking Reagent (BD, catalog number: 554714), and incubate 20 minutes for 4°C in the dark. After centrifugation at 150 ×g for 5 minutes at room temperature and washed twice with 1X Binding Buffer, phycoerythrin−labeled CD206 antibody (ThermoFisher) was then added to the cells and incubated in the dark for 30 minutes. Subsequently, all cells were then washed twice with 1X binding buffer and then and resuspended in 1X Binding Buffer. Finally, cells were analyzed using a BD FACSAria™ (III-type) flow cytometer (BD Biosciences). In addition, subsequent experiments were performed with mixed labeling of CD86 and CD206 by first labeling CD86 alone according to the method described above and then continuing to label CD206 as described above, with the experiments conducted under light-proof conditions throughout.

### 2.6 Enzyme-linked immunosorbent assay

The concentrations of NOS2, ARG1 and cytokines (TNF-α, IL1-β, TGF-β and IL10) in the extracted treatment and control RAW 264.7 culture supernatant samples were measured using ELISA kits (molbio) according to the manufacturer’s instructions. An automated microplate reader (Tecan, Sunrise, CH) was used to read the optical density at 450 nm.

### 2.7 Immunofluorescence analysis

Macrophages were pre-cultured in petri dishes with cover slides. *B. abortus* infection of macrophages for 12 hours as described in the “Cell Culture and Infection” section of the “Materials and Methods”. The cover slides were removed and washed twice with PBS. The cells were fixed with 4% paraformaldehyde at room temperature for 0.5 hours. PBS was washed twice and then permeabilized with 0.1% Triton X-100 for 0.5 hours. Permeabilized cells were washed twice with PBS and blocked with 1% BSA for 0.5 h at room temperature, followed by overnight incubation with anti-p-p65 (ab76302, 1:100) at 4°C. Wash twice with PBS containing 0.05% Tween-20 (PBST) and incubate for two hours at room temperature with Alexa Fluor™ 488 fluorescently labeled goat anti-rabbit IgG antibody (ab150077, 1:1000) protected from light. After wash twice with PBST, DAPI staining solution was added and incubated at room temperature and protected from light for 15 min. After twice PBST washes, images were collected using a confocal laser scanning microscope (Carl Zeiss 510, Germany).

### 2.8 Chromatin immunoprecipitation sequencing

An NF-κB ChIP-seq assay was conducted using a previously described method (26, 27). Briefly, RAW 264.7 were infected with S2308 (MOI = 100:1), while the control group was not infected. After the medium was incubated for 4 h, 1% formaldehyde was added to cross-link at room temperature for 15 minutes, then glycine to a final concentration of 125 mmol/L was added and the reaction was quenched at room temperature for 5 minutes. Discard the medium and wash the cells 2x with cold PBS. The PBS was discarded and then pre-chilled PBS containing a protease inhibitor mixture was added to the culture dish. The cells were collected with a cell scraper, centrifuged at 150 ×*g* and 4°C for 5 min, and the supernatant was discarded. The cells were then resuspended in 0.5 mL of cell lysis buffer containing the protease inhibitor mixture and incubated on ice for 15 min. The cells were centrifuged again at 150 ×g and 4°C for 5 min and the supernatant was discarded. Samples were then lysed by adding 0.5 ml of cell nuclear lysis buffer containing protease inhibitor cocktail and the chromatin was kept on ice. Sonicate chromatin to obtain soluble sheared chromatin (average DNA length 200-500 bp). An aspirate 100 μL of chromatin was obtained and incubated for 8 h at 4°C with 5 μg of NF-κB antibody (Abcam #19870) for immunoprecipitation. Subsequently, add 30 µL of protein beads to the sample tube and incubate at 4°C for 2 h. Wash once with 20mM Tris/HCl (pH 8.1), 50mM NaCl, 2mM ethylenediaminetetraacetic acid (EDTA), 1% Triton X-100, and 0.1% sodium dodecyl sulfate (SDS); Use twice with Tris/HCl (10mM, pH 8.1), LiCl (250mM), EDTA (1mM), NP-40 (1%), and deoxycholic acid (1%). Twice with TE buffer. The conjugate was then eluted with 250 μL of elution buffer (SDS (1%), NaHCO_3_ (100 mM)), treated with RNase A (8 μg/ml) at 65°C for 5 h, followed by proteinase K (345 μg treated/ml) overnight at 45°C. Immunoprecipitated DNA was constructed using the INEXTFLEX^®^ ChIP-Sew Library Prep Kit of the Illumina^®^ Sequencing Kit (NOVA-5143, Bio Scientific) according to the manufacturer’s protocol to create a sequencing library and then used the PE 150 method on the Illumina Xten platform Sequencing.

### 2.9 Bioinformatics analysis

Low-quality reads were filtered using Trimomatic (version 0.30) (28). The filtered clean reads were then mapped to the mouse genome using the Burrows Wheeler aligner (version 0.7.9). Calling peaks with parameters were using the Homer (version 4.8). The common peaks and unique peaks were identified by Homer use the tool mergePeaks with default parameters except -d 100. Peaks were assigned to genes when they located nearest to the gene’s transcription start site. Gene Ontology (GO) enrichment analysis was performed on the sample data using EasyGO (http://bioinformatics.cau.edu.cn/easygo/) (29). The P-value cut-off was set to 0.01, and the enrichment of the GO term was calculated using a hypergeometric distribution. False detection rates were calculated to adjust the P values for multiple comparisons after passing Fisher’s exact test with the aim of detecting overrepresented GO terms. To reveal the potential functions of the genes, Kyoto Encyclopedia of Genes and Genomes (KEGG) pathway (http://www.genome.jp/kegg/) enrichment analysis (30) was performed using the R package ClusterProfiler (http://www.bioconductor.org/packages.html) (31).

### 2.10 Construction of vectors and reporter gene assays

Full-length sequences of NF-κB *p65* and *Gls* genes were synthesized and subcloned into the pcDNA3.1 expression vector, respectively, to obtain stable pcDNA3.1-*p65* and pcDNA3.1-*Gls* overexpression plasmids. The full gene synthesis CMV base promoter and a 300 bp sequence predicted (**Supplementary Table 1B**) to be located in the intron region of the *Gls* gene were subcloned into the pGL3-basic vector polyclonal site to obtain pGL3-CMVmp-basic and pGL3-intron+CMVmp-basic. Cells were transfected with the overexpression plasmid and the firefly luciferase reporter gene plasmid with the Advanced DNA/RNA Transfection Reagent ™ (ZETA LIFE, USA). Renilla luciferase expression plasmid was used as a control for transfection efficiency to co-transfect with cells. After 48 hours of transfection, luciferase activity was measured using a dual-luciferase reporter system (Promega, Madison, USA) according to the manufacturer’s instructions.

### 2.11 Western blotting

Cells were collected 12h and 48h after *B. abortus* infection. The supernatant was extracted as the total protein by centrifugation with RIPA lysis buffer (Solarbio Science & Technology, #R0010, China) on ice for 0.5hours at 2000 ×*g*, and protein concentrations were determined using the BCA kit (Thermo Fisher Scientific). Proteins (20 μg) separated by 12% SDS-polyacrylamide gel electrophoresis were transferred to polyvinylidene fluoride membranes (Millipore, MA, USA) by a wet transfer system. Membranes were blocked with 5% (w/v) skimmed-milk for 4 hours at room temperature. Then incubated with the following primary-antibodies (Abcam, USA): anti-p-NF-κB (ab76302, 1:1000), anti-NF-κB (ab32536, 1:1000), anti-p-IKBα (ab133462, 1:1000), anti-IKBα (ab32518, 1:1000), anti-GLS (156876, 1:1000). The membrane was incubated with horseradish peroxidase-conjugated IgG overnight at 4°C and protein synthesis was analyzed using an enhanced chemiluminescence system. The relative expression of each protein normalized to the control β-actin was analyzed using Image J software.

### 2.12 Statistical analysis

The statistical analysis to determine the significance of differences between parameters was performed with GraphPad Prism 8 software using a one-tailed t-test. Data are expressed as mean values ± standard deviation (SD); p< 0.05 was considered statistical significance. Each treatment was repeated at least three times.

## 3 Results

### 3.1 *B. abortus* induces M1/M2-polarized phenotype transformation in RAW264.7 cells

**Figure 1:**
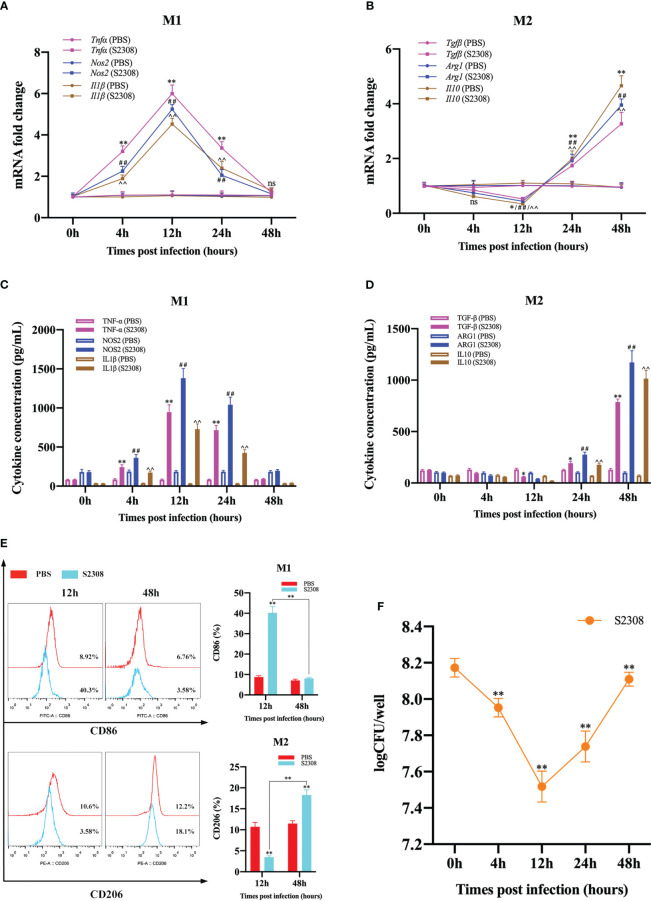
*B*. *abortus* induces M1/M2-polarized phenotype transformation in RAW264.7 cells. *B*. *abortus* infection (MOI = 100:1) of macrophage at different time points (0h, 4h, 12h, 24h and 48h). **(A, B)** RT-qPCR detection macrophage M1 (*Tnfα*, *Nos2* and *Il1β*) and M2 (*Tgfβ*, *Arg1*, *Il10*) polarization marker genes mRNA expression. **(C, D)** ELISA was performed to detect the secretion of M1 (TNF-α, NOS2, IL1β) and M2 (TGF-β, ARG1, IL10) related marker proteins in macrophage culture supernatants. **(E)** Flow cytometry to detect the expression of macrophage M1/M2 surface markers CD86/CD206 after 12 h and 48 h of *B*. *abortus* infection. **(F)** The number of *B*. *abortus* surviving in RAW264.7 cells after *B*. *abortus* infection at different time points. Data are shown as mean ± SD (n = 3). *p < 0.05, **p < 0.01; ^##^p < 0.01; ^^p < 0.01; one-tailed t-test; ns, not significant.

To investigate the effect of *B. abortus* infection on the M1/M2 polarization phenotype of RAW264.7 cells. Macrophages and supernatants were collected after different times (0h, 4h, 12h, 24h and 48h) of *B. abortus*-infection, and mRNA expression of macrophage M1 (*Tnfα*, *Nos2* and *Il1β*) and M2 (*Tgfβ*, *Arg1*, *Il10*) polarization marker genes were detected by RT-qPCR. ELISA was performed to detect the secretion of M1 (TNF-α, NOS2, IL1β) and M2 (TGF-β, ARG1, IL10)-related marker proteins in macrophage culture supernatants. The results showed that, compared with the control group, as the infection time increased, the expression of M1 marker genes and the secretion of M1 marker protein increased first and then decreased, these levels increased significantly at 4 h after infection, peaked at 12 h, and then decreased gradually thereafter up to 48 h (**Figures 1A, C** **)**. Expression of M2 marker genes and secretion of M2 marker proteins, on the other hand, were not expressed or decreased initially in infection and then increased during infection; the lowest levels were observed at 12 h, prior to undergoing significant increases (**Figures 1B, D** **)**. Further analysis by flow cytometry showed that *B. abortus* infection at 12 hours showed a significant upregulation of the expression of the M1-type marker CD86 and the M2-type marker CD206 was significantly reduced. However, no expression of CD86 was induced at 48 hours of infection, but CD206 expression was significantly upregulated (**Figure 1E**). In addition, we analyzed the intracellular survival of *B. abortus* infection macrophages at different times and showed that the intracellular bacterial Colony-Forming Unit (CFU) values first gradually decreased with increasing time of infection, reaching a trough at 12 hours and then gradually increasing (**Figure 1F**). Thus, these results suggest that *B. abortus* infection induces a dynamic shift in macrophage phenotype from M1 to M2, thereby favoring their intracellular survival.

### 3.2 *B. abortus* infection induces altered activation state of NF-κB Signaling pathway in RAW264.7 cells

**Figure 2:**
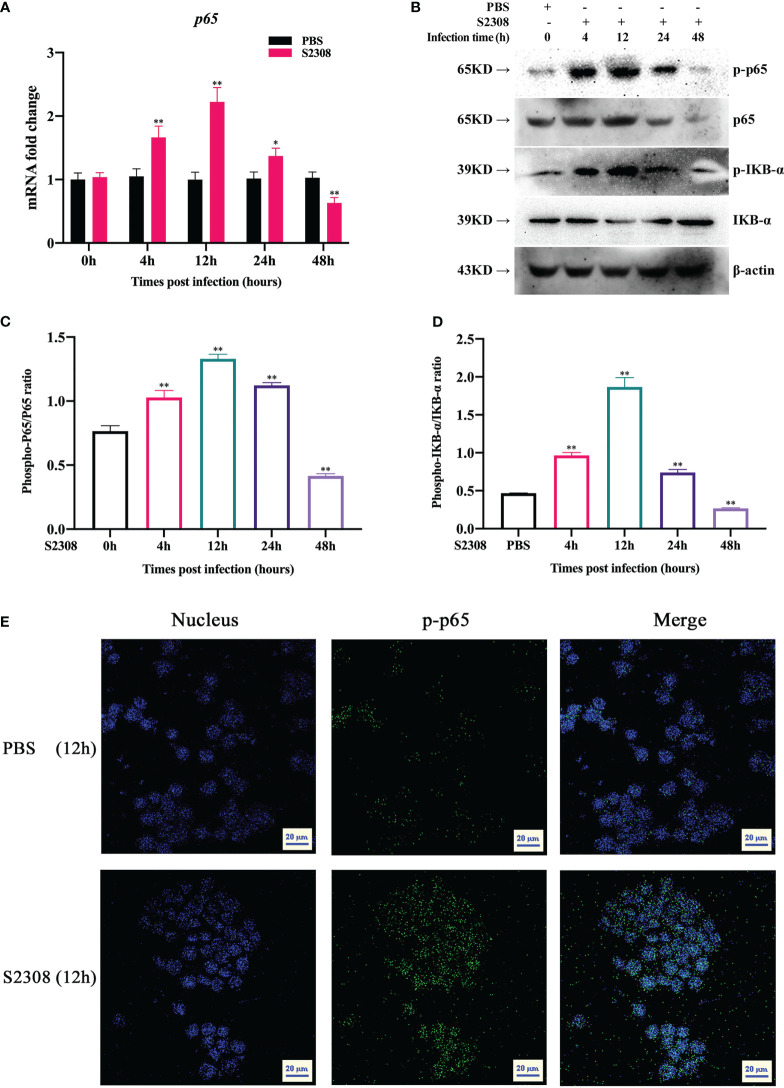
*B*. *abortus* infection induces altered activation state of NF-κB signaling pathway in RAW264.7 Cells. **(A)** RT-qPCR to detect the expression of NF-kB *P65* mRNA levels in *B*. *abortus*-infected RAW 264.7 cells at different time points (0h, 4h, 12h, 24h and 48h). **(B–D)** Western blotting to determine phosphorylation of IκBα and p65 protein levels in *B*. *abortus*-infected RAW 264.7 cells at different time points (0h, 4h, 12h, 24h and 48h). **(E)** *B*. *abortus*-infected RAW 264.7 cells for 12 h. PBS was used as a control and immunofluorescence staining was performed for p-p65 (green). DAPI (blue) were used to stained nuclei. Scar bar: 20 μm. Data are shown as mean ± SD (n = 3). *p < 0.05, **p < 0.01; one-tailed t-test; ns, not significant.

NF-κB is a key transcriptional regulator that regulates the polarization of M1/M2 macrophages. To better determine the activation of NF-κB signaling pathway at different time periods (0h, 4h, 12h, 24h and 48h) in *B. abortus* infection RAW264.7 macrophages, key genes and proteins of the signaling pathway were examined. The results of RT-qPCR showed that the expression of p65 mRNA level in the *B. abortus* infection group gradually increased from 0 to 12 h, reached a peak at 12 h, and then decreased (**Figure 2A**). Western blot analysis also showed that IκBα and p65 were phosphorylated after *B. abortus* infection group first increased, reached a peak at 12 h, and then decreased (**Figures 2B-D** **)**. The expression trends of P65 phosphorylation were consistent with the expression trends of M1-associated inflammatory genes and cellular supernatant M1-associated secretory proteins after different times of *B. abortus* infection. Further, we examined the fluorescence intensity of P-P65 protein after 12 h of Brucella infection of RAW264.7 cells using laser scanning confocal microscope. The results showed that the fluorescence intensity of P-P65 protein was significantly enhanced in the *B. abortus* infection group compared with the PBS control group, and the fluorescence intensity of P-P65 protein localized in the nucleus (blue) was significantly increased (**Figure 2E**). Thus, these results suggest that *B. abortus* can induce a shift from an activated to an inhibited activation state of the NF-κB signaling pathway in RAW264.7 cells. This suggests that the NF-κB signaling pathway may be involved in *B. abortus*-induced regulation of macrophage polarization.

### 3.3 *B. abortus* regulates the M1/M2 polarization phenotype of macrophages through the NF-κB signaling pathway

To further determine whether *B. abortus* regulates macrophage polarization through the NF-κB signaling pathway. Macrophages were pretreated with a chemical inhibitor (BAY) of the NF-κB signaling pathway for 1h or transfected with NF-κB overexpression plasmids (pCDNA3.1-*p65*) for 12 h. RT-qPCR, Western blot, ELISA and Western blot method was used to detect the activation of NF-κB after 12h and 48h of *B. abortus* infection as well as the expression of cell polarization-related marker genes, secretion of culture fluid-associated marker proteins and the ratio of cell polarization surface markers CD86 and CD206. PBS was used as a control, in addition, LPS (100 ng/ml) and IL4 (10 ng/ml) stimulated macrophages were used as positive controls to induce M1 and M2 polarization. The CCK8 method was used to determine the optimal action concentration of BAY.

**Figure 3:**
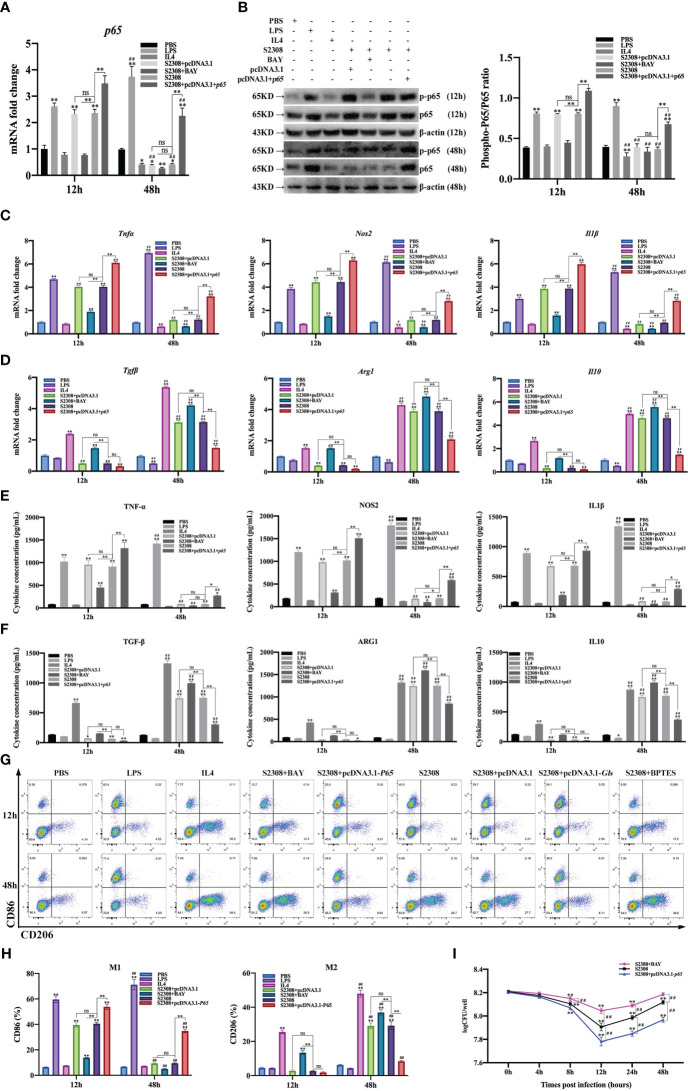
*B*. *abortus* regulates the M1/M2 polarization phenotype of macrophages through the NF-κB signaling pathway. Macrophages were pretreated with a chemical inhibitor (BAY, 1μM) of the NF-κB signaling pathway for 1h or transfected with NF-κB overexpression plasmids (pCDNA3.1-p65) for 12 h and used for *B*. *abortus* infection. The pretreated macrophages were infected with *B*. *abortus* for 12 h and 48 h. PBS was used as a control, in addition, LPS (100 ng/ml) and IL4 (10 ng/ml) stimulated macrophages were used as positive controls to induce M1 and M2 polarization. **(A)** RT-qPCR to detect the expression of NF-kB *P65* mRNA levels in macrophages of each group. **(B)** Western blotting to determine phosphorylation of p65 protein levels in macrophages of each group. **(C, D)** RT-qPCR detection macrophage M1 (*Tnfα*, *Nos2* and *Il1β*) and M2 (*Tgfβ*, *Arg1*, *Il10*) polarization marker genes mRNA expression. **(E, F)** ELISA was performed to detect the secretion of M1 (TNF-α, NOS2, IL1β) and M2 (TGF-β, ARG1, IL10) related marker proteins in macrophage culture supernatants. **(G, H)** Flow cytometry to detect the expression of macrophage M1/M2 surface markers CD86/CD206 in macrophages of each group. **(I)** The number of *B*. *abortus* surviving in RAW264.7 cells of each group after *B*. *abortus* infection at different time points (0h, 4h, 8h, 12h, 24h and 48h). Data are shown as mean ± SD (n = 3). *p < 0.05, **p < 0.01; Compared with 12h, ^##^p < 0.01; one-tailed t-test; ns, not significant.

CCK-8 results showed that 1 μM BAY treatment had no effect on the activity of RAW264.7 cells; therefore, 1 μM BAY was selected for subsequent experiments (**Supplementary Figure 1A**). RT-qPCR and Western blot results showed that *p65* mRNA and phosphorylated p65 protein expression were significantly increased in the early phase of *B. abortus* infection (12 h) and significantly decreased in the late phase of infection (48 h) compared to the PBS group. BAY significantly inhibited *p65* mRNA and phosphorylated p65 protein expression early in infection, but there was no difference in late infection. Overexpression of the *p65* gene significantly promoted *p65* mRNA and phosphorylated p65 protein expression in both the early and late stages of infection. *B. abortus* induced significantly higher expression of *p65* mRNA and phosphorylated p65 protein early in the infection than late in the infection. The effect of LPS on macrophages at both 12h and 48h promoted *p65* mRNA and phosphorylated p65 protein expression and gradually increased with time. IL4 effect on macrophages at 48h significantly inhibited *p65* mRNA and phosphorylated p65 protein expression (**Figures 3A, B** **)**.

RT-qPCR **(** **Figures 3C, D** **)**, ELISA (**Figures 3E, F** **)**, and flow cytometry (**Figures 3G, H** **)** analyses showed that the expression of M1-type marker gene mRNA, secretion of M1-type marker protein, and expression of M1-type surface marker CD86 (expression of M1-type-associated markers) were significantly increased in the early stages of *B. abortus* infection compared with the PBS group, whereas the differences were not significant in the late stages of infection. In contrast, the expression of M2-type-associated markers tended to decrease or not be expressed early in *B. abortus* infection, whereas it increased significantly late in the infection. BAY significantly inhibited the expression of M1-type-associated markers in the early stage of infection, and partially inhibited or differed insignificantly in the late stage of infection. In addition, it significantly promoted the expression of M2-type-related markers in both the early and late stages of infection. Overexpression of the p65 gene significantly promoted the expression of M1-type-associated markers at both early and late stages of infection, with non-significant differences in the expression of M2-type-associated markers at early stages of infection, but significant inhibition at late stages of infection. *B. abortus* induced significantly higher expression of M1-type-associated markers early in the infection than late in the infection, while M2-associated markers were significantly less expressed than late in the infection. LPS action on macrophages promoted the expression of M1-type-related markers at both 12h and 48h and gradually increased with time. In contrast, M2-associated markers were partially inhibited or not significantly expressed at 12h and 48h. The expression of M1-type-related markers was partially inhibited or not significantly expressed by IL4 acting on macrophages for 12h and 48h; however, the expression of M2-type-related markers was strongly induced and gradually increased with time.

In addition, intracellular CFU analysis was also performed on *B. abortus*-infected macrophages pretreated with BAY or overexpression of the *p65* gene (**Figure 3I**). The results showed that the CFU values of bacteria surviving in *B. abortus* infected macrophages tended to decrease and then increase, reaching a trough at 12 h and gradually increasing thereafter. At 12, 24 and 48 h of infection, BAY significantly increased CFU values and overexpression of the p65 gene significantly suppressed CFU values compared to *B. abortus* infection controls. Overall, these results suggest that *B. abortus* can regulate the dynamic shift in macrophage phenotype from M1 to M2 through the NF-κB signaling pathway. It further confirms that NF -κ B is a key pathway regulating the M1/M2 phenotype shift.

### 3.4 Functional analysis of NF-κB regulatory genes during *B. abortus* infection

**Figure 4:**
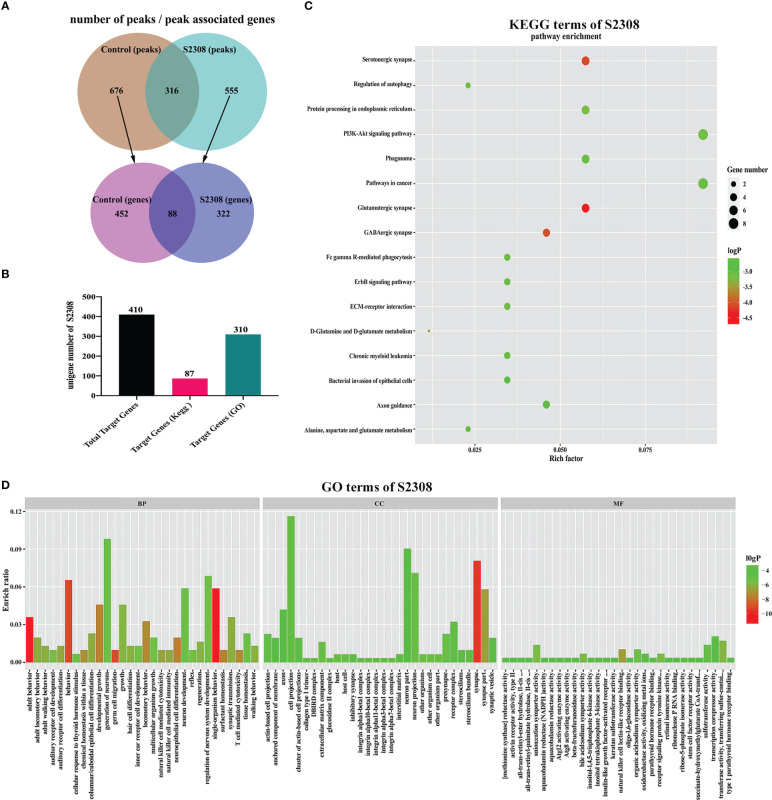
ChIP-seq analysis of the NF-κB target gene. **(A)** The number of peaks and peak associated genes in the control and S2308 groups. **(B)** The number of binding genes annotated to the KEGG pathway and GO terms in the S2308 infection group. **(C)** Top 16 enriched KEGG pathways for S2308-treated cells for 4 h. The X-axis represents the rich facto, and the Y-axis represents the pathway. **(D)** GO annotation classification results of the binding genes. The X-axis represents the GO functional classification and the Y-axis represents the rich factor.

We used ChIP-seq technology to functionally analyze genes regulated by NF-κB during *B. abortus* infection. The raw FASTQ sequencing data have been uploaded to the SRA database (PRJNA832890 (SRA, Release date: 2026-04-28)). As shown in **Figure 4A**, the Homer tool identified 992 peaks in the control group and 871 peaks in the S2308 group. The mergePeaks tool identified 676 unique peaks in the control group, 555 unique peaks in the S2308 group and 316 common peaks both the infected and control groups. Detailed genomic location information for these peaks is shown in **Supplementary Table 2**.** **A summary analysis of all peak-associated genes showed that 676 unique peaks in the control group associated 540 genes and 555 unique peaks in the S2308 group associated 410 genes. Of these, 88 genes were common to both the control and S2308 groups. In addition, details of all peaks in the control and S2308 groups, including associated genes, and genomic location information are shown in **Supplementary Tables 3**, **4**. As shown in **Figure 4B**, KEGG and GO analysis annotated 87 and 310 of the 410 binding genes in the S2308 group, respectively. The KEGG enrichment results are shown in **Figure 4C**. The “Rich factor” refers the ratio of the number of differentially expressed genes annotated to all genes annotated in the same path, with a greater Rich ratio indicating greater intensity of enrichment. The logP value is the corrected p value; the lower the value, the greater the intensity. KEGG analysis shows that the top 16 major enriched pathways for S2308 group included the glutamatergic synapse and phagosome signaling pathways, with five binding genes, respectively. GO analysis showed that these binding genes were affected by NF-κB. In the three categories of biological processes, cellular components, and molecular functions, there were 90 functional groups, among which the number of biological process genes was the largest, and the number of neuron generation genes was the largest (containing 30 genes). The cellular component category included 13 functional groups, and cell projection was the largest group in this category (containing 36 genes). The molecular functional classification also includes 13 functional groups, the largest of which was transcription corepressor activity (containing 6 genes) (**Figure 4D**).

### 3.5 Further screening and validation of key target genes of NF-κB-regulated polarization during *B. abortus* infection

**Figure 5:**
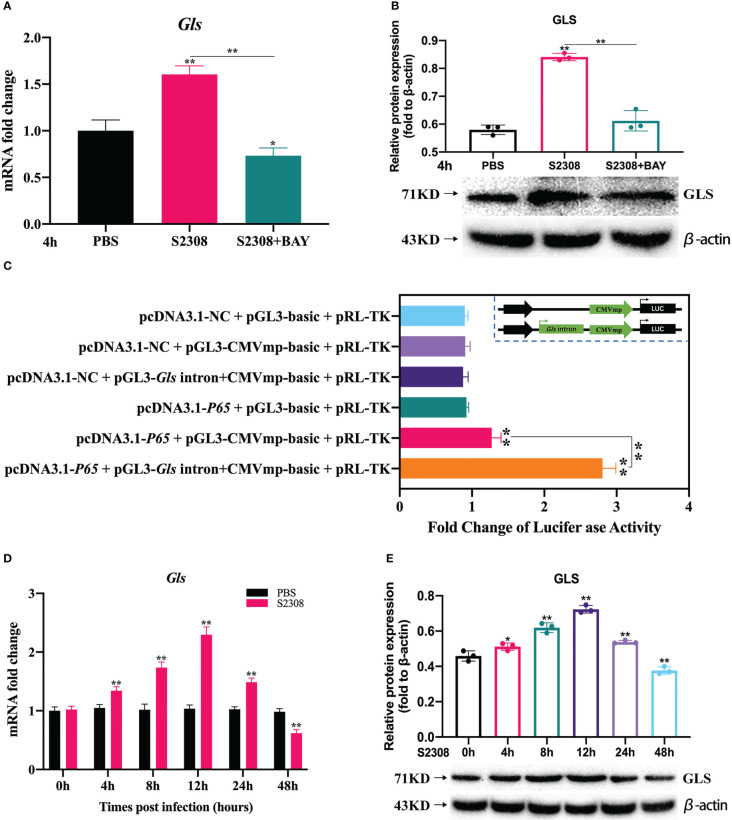
Further screening and validation of key target genes of NF-κB-regulated polarization during *B*. *abortus* infection. Macrophages were pretreated with BAY (1μM) for 1h used for **(B)** abortus infection. The pretreated macrophages were infected with **(B)** abortus for 4h, normal cells were used as a control group. **(A)** Verification of *Gls* regulated by NF-kB using RT-qPCR. **(B)** Verification of GLS regulated by NF-kB using western blot. **(C)** Dual luciferase reporter assays of pcDNA3.1-*p65* in response to pGL3-CMVmp-basic and pGL3-*Gls* intron+CMVmp-basic. *B*. *abortus*-infected RAW 264.7 cells at different time points (0h, 4h, 8h,12h, 24h and 48h). **(D)** RT-qPCR to detect the expression of *Gls* mRNA levels. **(E)** Western blotting to determine GLS protein levels. Data are shown as mean ± SD (n = 3). *p < 0.05, **p < 0.01; one-tailed t-test.

For the genes annotated by KEGG and GO analysis, on the basis of excluding 88 common genes, we focused on the genes located in introns, promoter-TS, non-coding 5′ untranslated regions (UTRs), 3′ UTRs, exons, transcription start sites, and other specific regions. Using website predictions and combined with relevant literature reports, we screened the *Gls* gene as a possible target gene for NF-κB-regulated polarization. RT-qPCR, dual luciferase reporter assays and Western blot were used to verify the targeting relationship between NF-κB and *Gls*, by examining the expression of *Gls* at different times of *B. abortus* infection to assess its potential as a key target gene for regulating M1/M2 polarization. The results showed that the *B. abortus* infection group strongly induced GLS mRNA and protein expression and BAY significantly inhibited GLS mRNA and protein expression compared with the PBS group (**Figures 5A, B** **)**. The CMV basic promoter is a piece of universal promoter sequence containing multiple NF-κB binding sites. Full gene synthesis CMVmp and *Gls* intron+CMVmp sequences were synthesized and subcloned into pGL3-basic to obtain reporter gene plasmids, respectively, which were cotransfected in macrophages with overexpressed NF-κB p65 plasmid. The luciferase assay showed that luciferase activity was enhanced in the pcDNA3.1-*p65* + pGL3-CMVmp-basic group compared to the pcDNA3.1-*p65* + pGL3-basic group, while luciferase activity was further enhanced in the pcDNA3.1-*p65* + pGL3-*Gls* intron+CMVmp-basic (**Figure 5C**). In addition, GLS mRNA and protein expression gradually increased early in Brucella infection (0-12 h), peaked at 12 h, and gradually decreased later in the infection (12-48 h) (**Figures 5D, E** **)**. The trend of GLS mRNA and protein expression was consistent with the trend of Brucella-induced expression of M1-type-related markers. These results suggest that NF-κB enhances *Gls* gene expression by binding to the predicted NF-κB binding sequence in the first intron of the *Gls* gene. *Gls* is potentially involved in the regulation of Brucella-induced macrophage polarization.

### 3.6 *B. abortus* regulates the M1/M2 polarization phenotype of macrophages through *Gls*, a NF-κB signaling pathway target gene

To further determine whether *B. abortus* regulates macrophage polarization through the NF-κB signaling pathway target gene *Gls*. Macrophages were pretreated with a chemical inhibitor (BPTES) of the GLS for 1h or transfected with *Gls* overexpression plasmids (pCDNA3.1-*Gls*) for 12 h. RT-qPCR, Western blot, ELISA and Western blot method was used to detect the activation of NF-κB p65 and Gls after 12h and 48h of *B. abortus* infection as well as the expression of cell polarization-related marker genes, secretion of culture fluid-associated marker proteins and the ratio of cell polarization surface markers CD86 and CD206. PBS was used as a control, in addition, LPS (100 ng/ml) and IL4 (10 ng/ml) stimulated macrophages were used as positive controls to induce M1 and M2 polarization. The CCK8 method was used to determine the optimal action concentration of BPTES.

**Figure 6:**
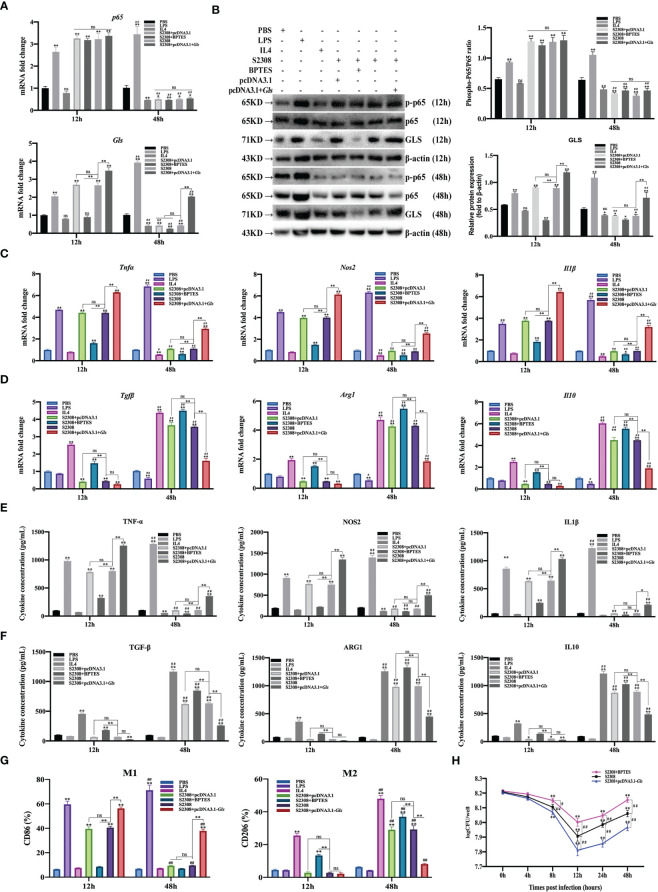
*B*. *abortus* regulates the M1/M2 polarization phenotype of macrophages through *Gls*, a NF-κB signaling pathway target gene. Macrophages were pretreated with a *Gls* chemical inhibitor (BPTES, 5μM) for 1h or transfected with *Gls* overexpression plasmids (pCDNA3.1-*Gls*) for 12h and used for *B*. *abortus* infection. The pretreated macrophages were infected with *B*. *abortus* for 12 h and 48 h. PBS was used as a control, in addition, LPS (100 ng/ml) and IL4 (10 ng/ml) stimulated macrophages were used as positive controls to induce M1 and M2 polarization. **(A)** RT-qPCR to detect the expression of NF-kB *P65* and *Gls* mRNA levels in macrophages of each group. **(B)** Western blotting to determine GLS and phosphorylation of p65 protein levels in macrophages of each group. **(C, D)** RT-qPCR detection macrophage M1 (*Tnfα*, *Nos2* and *Il1β*) and M2 (*Tgfβ*, *Arg1*, *Il10*) polarization marker genes mRNA expression. **(E, F)** ELISA was performed to detect the secretion of M1 (TNF-α, NOS2, IL1β) and M2 (TGF-β, ARG1, IL10) related marker proteins in macrophage culture supernatants. **(G)** Flow cytometry to detect the expression of macrophage M1/M2 surface markers CD86/CD206 in macrophages of each group. **(H)** The number of *B*. *abortus* surviving in RAW264.7 cells of each group after *B*. *abortus* infection at different time points (0h, 4h, 8h, 12h, 24h and 48h). Data are shown as mean ± SD (n = 3). *p < 0.05, **p < 0.01; Compared with 12h, ^#^p < 0.05, ^##^p < 0.01; one-tailed t-test; ns, not significant.

CCK-8 results showed that 5 μM BPTES treatment had no effect on the activity of RAW264.7 cells; therefore, 5 μM BPTES was selected for subsequent experiments (**Supplementary Figure 1B**). RT-qPCR and Western blot results showed that *p65* and *Gls* related mRNAs and proteins expression were significantly increased in the early phase of *B. abortus* infection (12 h) and significantly decreased in the late phase of infection (48 h) compared to the PBS group. BPTES significantly inhibited *Gls* mRNA and protein expression early in infection, but there was no difference in late infection. Overexpression of the *Gls* gene significantly promoted *Gls* mRNA and protein expression in both the early and late stages of infection. BPTES or overexpression of the *Gls* gene had no effect on *p65* mRNA and phosphorylated protein expression. *B. abortus* induced significantly higher expression of *Gls* mRNA and protein early in the infection than late in the infection. The effect of LPS on macrophages at both 12h and 48h promoted *Gls* mRNA and protein expression and gradually increased with time. IL4 effect on macrophages at 48h significantly inhibited *Gls* mRNA and protein expression (**Figures 6A, B** **)**.

RT-qPCR **(** **Figures 6C, D** **)**, ELISA (**Figures 6E, F** **)**, and flow cytometry (**Figure 6G**) analyses showed that the expression of M1-type marker gene mRNA, secretion of M1-type marker protein, and expression of M1-type surface marker CD86 (expression of M1-type-associated markers) were significantly increased in the early stages of *B. abortus* infection compared with the PBS group, whereas the differences were not significant in the late stages of infection. In contrast, the expression of M2-type-associated markers tended to decrease or not be expressed early in *B. abortus* infection, whereas it increased significantly late in the infection. BPTES significantly inhibited the expression of M1-type-associated markers in the early stage of infection, and partially inhibited or differed insignificantly in the late stage of infection. In addition, it significantly promoted the expression of M2-type-related markers in both the early and late stages of infection. Overexpression of the *Gls* gene significantly promoted the expression of M1-type-associated markers at both early and late stages of infection, with non-significant differences in the expression of M2-type-associated markers at early stages of infection, but significant inhibition at late stages of infection. *B. abortus* induced significantly higher expression of M1-type-associated markers early in the infection than late in the infection, while M2-associated markers were significantly less expressed than late in the infection. LPS action on macrophages promoted the expression of M1-type-related markers at both 12h and 48h and gradually increased with time. In contrast, M2-associated markers were partially inhibited or not significantly expressed at 12h and 48h. The expression of M1-type-related markers was partially inhibited or not significantly expressed by IL4 acting on macrophages for 12h and 48h; however, the expression of M2-type-related markers was strongly induced and gradually increased with time.

In addition, intracellular CFU analysis was also performed on *B. abortus*-infected macrophages pretreated with BPTES or overexpression of the *Gls* gene (**Figure 6H**). The results showed that the CFU values of bacteria surviving in *B. abortus* infected macrophages tended to decrease and then increase, reaching a trough at 12 h and gradually increasing thereafter. At 12, 24 and 48 h of infection, BPTES significantly increased CFU values and overexpression of the *Gls* gene significantly suppressed CFU values compared to *B. abortus* infection controls.

Overall, these results suggest that *B. abortus* can regulate the dynamic shift in macrophage phenotype from M1 to M2 through *Gls*, a NF-κB Signaling Pathway Target Gene. It further confirms that *Gls* is a key target gene in the NF-κB-regulated M1/M2 phenotype shift.

## 4 Discussion

Macrophages typically exhibit plasticity between the M1 and M2 phenotypes and the two phenotypes can be interconverted under different stimuli or microenvironments (32). There has been evidence that a variety of intracellular pathogens, including *Mycobacterium tuberculosis* and *Brucella*, can bias macrophages toward M1 or M2 phenotype, but the exact mechanisms are not well understood (6, 33). *B. abortus* is an intracellular parasitic bacterium that infects humans and animals and is highly pathogenic. *B. abortus* primarily invades host macrophages and replicates in them, thereby causing persistent infection (34). In this study, we found that *B. abortus* infection caused macrophages to switch from a pro-inflammatory M1 phenotype to an anti-inflammatory M2 phenotype, which was closely related to the release of inflammatory cytokines. During the early phase of infection (0-12h), *B. abortus* differentially promoted the expression of M1 marker genes and the secretion of M1 marker proteins, while M2 marker genes and proteins were not expressed or down-regulated, and the number of intracellularly surviving *B. abortus* gradually decreased. We hypothesize that at this stage, macrophages recognize *B. abortus* and polarize to the M1 phenotype, at which point large amounts of pro-inflammatory cytokines are released and the Th1 immune response is initiated. These M1 macrophages can eliminate *B. abortus via* phagocytosis. With the increase of infection time, at the late stage of infection (12-48h), *B. abortus* differentially suppressed the expression of M1 marker genes and the secretion of M1 marker proteins, while the M2 marker genes and proteins were significantly up-regulated and the number of intracellularly surviving *B. abortus* gradually increased. Therefore, we speculate that at this stage, a few intracellularly surviving *B. abortus* initiate a Th2 immune response by modulating the conversion of macrophages to an M2 polarized phenotype, inducing the release of large amounts of anti-inflammatory cytokines from macrophages, thereby avoiding clearance of the bacteria by the immune system and promoting their intracellular survival. Our findings are consistent with previous reports (6). Thus, these results suggest that Brucella infection induces a shift in macrophage phenotype from the M1 to the M2 phenotype, thereby facilitating their intracellular survival.

Previous reports have shown that activated NF-κB and NF-κB-mediated antibacterial immunity are related to an inflammatory response in *B. abortus* infection (35, 36). There is growing evidence that NF-κB plays a vital role in regulating polarization and inflammation (37). However, the effect of the NF-κB signaling pathway on cell polarization in the process of *B. abortus* infecting macrophages are unclear. Therefore, in this study, we first analyzed and detected the expression of several key genes and the phosphorylation levels of several key proteins in the NF-κB signaling pathway at different times of *B. abortus* infection. It was showed that the expression of the *P65* gene and the phosphorylation levels of P65 and IκBα gradually increased during the early stages of *B. abortus* infection, peaking at 12 h, and gradually decreased during the later stages of infection. The expression trends were consistent with those of M1-associated inflammatory genes and cell supernatant M1-associated secretory proteins after different times of *B. abortus* infection of macrophages. In addition, at 12h of *B. abortus* infection, we observed a significant enhancement of fluorescent labeling of cells with p-p65 protein in the nucleus. To further determine the role of NF-κB signaling pathway in *B. abortus*-induced polarization phenotype switching in macrophages, we blocked or activated this signaling pathway by means of NF-κB inhibitors and NF-κB overexpression plasmids. Macrophages were induced to M1 and M2 phenotypes by stimulation with LPS and IL4, respectively, as positive controls. The results showed that NF-κB inhibitors differentially down-regulated the expression of M1 related markers, up regulated the expression of M2 related markers, and the number of intracellularly viable *B. abortus* increased. In contrast, overexpression of NF-κB differentially upregulated the expression of M1 related markers and downregulated the expression of M2 associated markers, and the number of intracellularly viable *B. abortus* was reduced. In addition, we found that NF-κB inhibitors and overexpressed NF-κB acted differently degrees on M1/M2-related markers at different times (12h and 48h) of *Brucella* infection, which may be caused by inconsistent basal expression of NF-κB at 12h and 48h of *Brucella* infection. Overall, these results suggest that *B. abortus* can regulate the dynamic shift in macrophage phenotype from M1 to M2 through the NF-κB signaling pathway. It further confirms that NF -κ B is a key pathway regulating the M1/M2 phenotype shift.

The transcription factor NF-κB plays a central role in regulating inflammation, cell differentiation, and Th1/Th2 immunity through its target genes (12, 38). However, it is difficult to accurately identify the direct target of NF-κB with respect to the regulation of cell polarization in the process of *B. abortus* infection. In the present study, the ChIP-seq results showed that NF-κB had a high *Gls*-binding activity. Recent studies have shown that glutamine plays a vital role in M1 macrophages induced by Mycobacterium tuberculosis infection (39), and in addition, glutamine is important for LPS-induced activation of mTOR1C in M1 macrophages (40, 41). However, several other studies have showed that glutamine metabolism limits the pro-inflammatory M1 state in favor of M2 polarization (42–44). The definitive role of glutamine in infection-induced macrophage polarization has not been determined due to conflicting reports, particularly during *B. abortus* infection. *Gls*, a key enzyme in glutamine catabolism, is likely to play a key role in the regulation of *B. abortus*-induced cell polarization (45–47). Our results show that NF-κB inhibitors significantly inhibit GLS mRNA and protein expression. NF-κB enhances *Gls* gene expression by binding to the predicted NF-κB binding sequence in the first intron of the *Gls* gene. The expression trends of GLS mRNA and protein were consistent with the expression trends of M1-type related markers induced after different times of *B. abortus* infection. Further studies showed that *Gls* inhibitors differentially downregulate the expression of M1-associated markers and upregulate the expression of M2-associated markers, and the number of intracellularly viable *B. abortus* increased. In contrast, overexpression of *Gls* differentially upregulated the expression of M1-related markers and downregulated the expression of M2-associated markers, and the number of intracellularly viable *B. abortus* was reduced. In addition, GLS and phosphorylated P65 protein expression were reduced in *B. abortus* infection-induced macrophages at 48h, and overexpression of *Gls* significantly reversed M1 inhibition and M2 promotion by B. abortus infection. Overall, these results indicate that B. abortus can regulate the dynamic shift in macrophage phenotype from M1 to M2 through *Gls*, a NF-κB Signaling Pathway Target Gene. These findings further suggest that *Gls* plays an important role in *Brucella*-mediated M1/M2 phenotypic transformation and anti-*Brucella* drug treatment.

**Figure 7:**
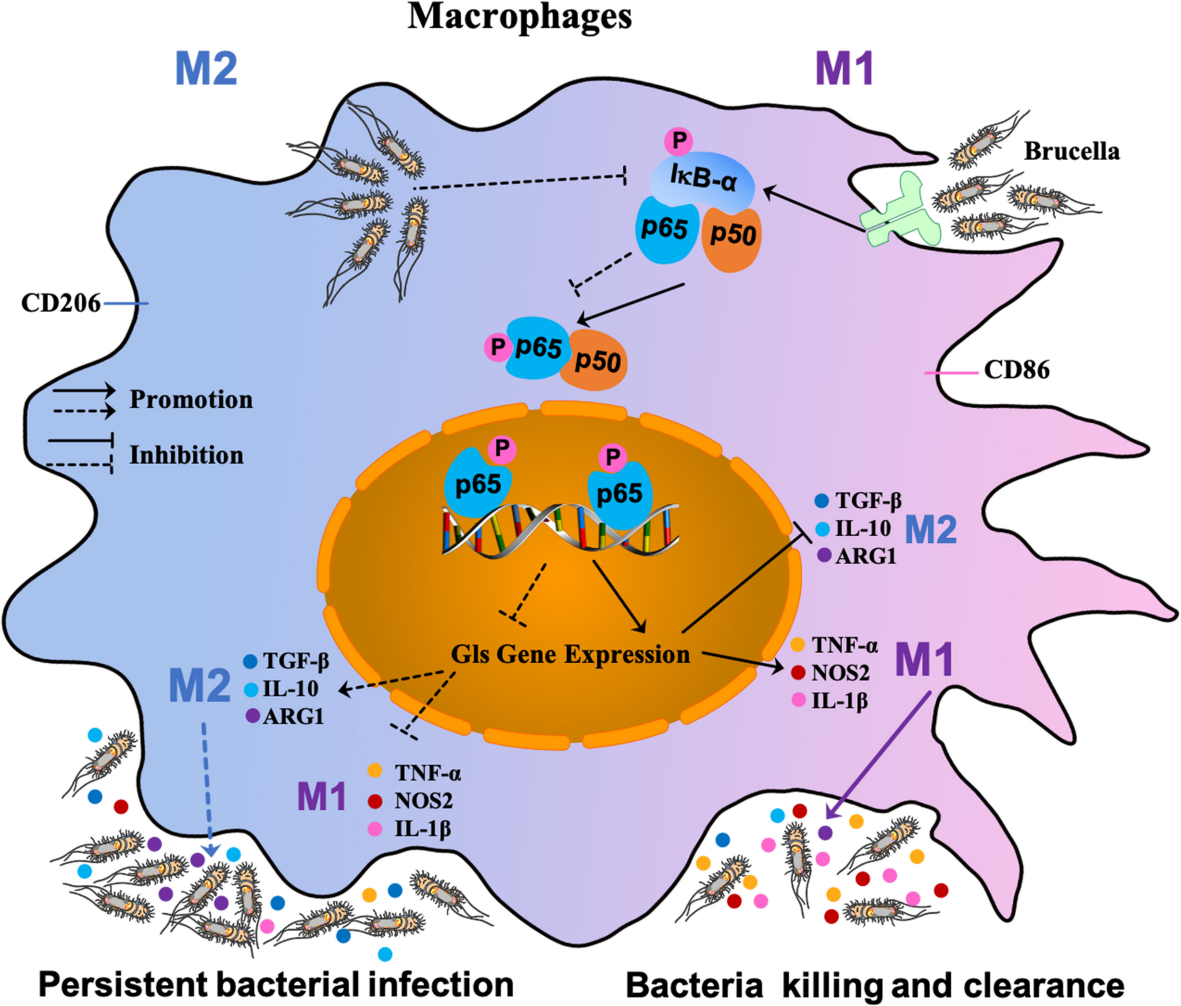
Schematic diagram of S2308-infected macrophages. Recognition of PAMPs of *B. abortus* by TLRs on the surface of host macrophages triggers a cascade reaction in which, on the one hand, activation of IκB-α leads to phosphorylation of the cascade’s P65 into the nucleus, thereby inducing the expression of the target gene *Gls*. This cascade leads to a shift of macrophages towards an M1-polarized phenotype and the release of large amounts of pro-inflammatory factors that contribute to the clearance of *Brucella*. On the other hand, with prolonged infection, the few B. abortus that survive intracellularly suppress *Gls* expression by inhibiting P65 phosphorylation of the IκB-α cascade, causing macrophages to shift to the M2 phenotype, at which point large amounts of inflammatory suppressors are released to ensure their survival in the host.

Our results provide a general model for recognition of pathogen-associated molecular patterns (PAMPs) of *B. abortus* by toll-like receptors (TLRs) on the surface of host macrophages, which triggers a cascade response leading to macrophage polarization (48). TLRs recognition of *B. abortus* PAMPs triggering a cascade leading to the phosphorylation of IκB-α, which in turn leads to the release of NF-κB P65 and P50. The P65 protein is activated by phosphorylation and transported into the nucleus from the cytoplasm, where it induces the expression of the target gene *Gls*. This cascade results in the transformation of macrophages to the M1 polarization phenotype; a large number of pro-inflammatory factors (TNF-α, NOS2, and IL-1β) are then released that contribute to clearing *Brucella*. With prolonged infection, the few *B. abortus* that survive intracellularly suppress *Gls* expression by inhibiting phosphorylation of IκB-α and thus P65 phosphorylation and entry into the nucleus, and shifts macrophages to the M2 phenotype, releasing large amounts of inflammatory inhibitory factors such as TGF-β, ARG-1 and IL-10 to ensure its survival in the host (**Figure 7**).

In conclusion, our study shows that *B. abortus* infection induces a dynamic shift in the M1/M2 phenotype of macrophages. The mechanism involved is related to regulating the expression of NF-κB and its target gene *Gls*. This study is the first to elucidate the molecular mechanism by which *B. abortus* targets glutaminase through the NF-κB signaling pathway and thereby regulates macrophage polarization phenotype transformation and the inflammatory response. *Gls*, as a key target for regulating cell polarization, inflammation, and the immune response induced by *B. abortus* infection, should thus be the focus of future drug development and for designing molecular strategies based on macrophage polarization regulation. As such, these results have important implications for the treatment and prevention of *B. abortus* infection.

## Data availability statement

The datasets presented in this study can be found in online repositories. The names of the repository/repositories and accession number(s) can be found below: PRJNA832890 (SRA, Release date: 2026-04-28).

## Author contributions

TZ, HZ and ZZ designed the study. YL, LL and ZS were responsible for the acquisition and collection of data. XD and DZ contributed to the analysis and interpretation of data. JG and SC performed the bioinformatic analysis. YC prepared figures and reagents. UN and SM were involved in manuscript preparation. ZW and HZ provided approval of the final version to be published. All authors contributed to the article and approved the submitted version.
