# Supplementary figures and images for: Brucella abortus modulates macrophage polarization and inflammatory response by targeting glutaminases through the NF-κB signaling pathway
Source: Front Immunol. 2023 May 31;14:1180837. doi: 10.3389/fimmu.2023.1180837 (PMC10266586; doi:10.3389/fimmu.2023.1180837)

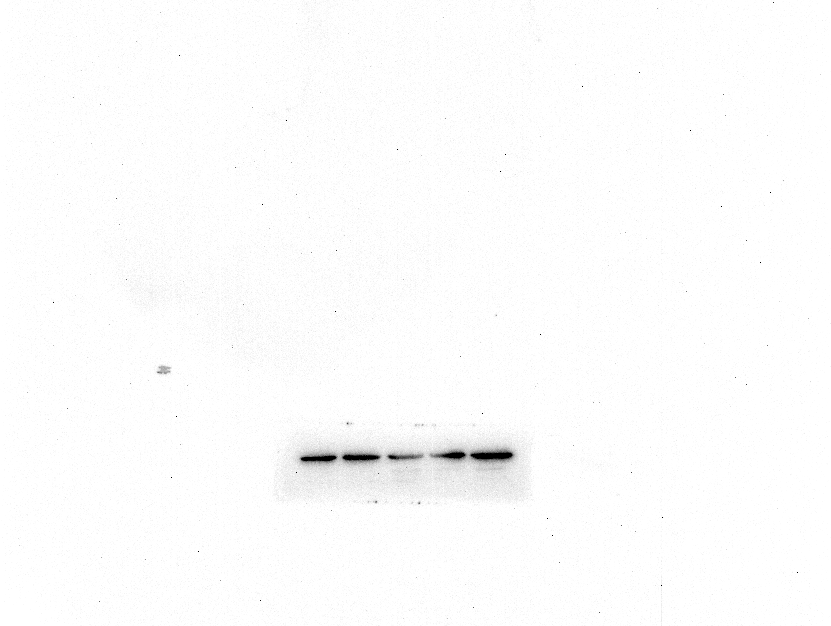

Supplement: Supplementary file 1 [file DataSheet_1.zip › Raw Date-1/Figure2/Figure2B/Western blot original images/IKB-α(39KD).png]

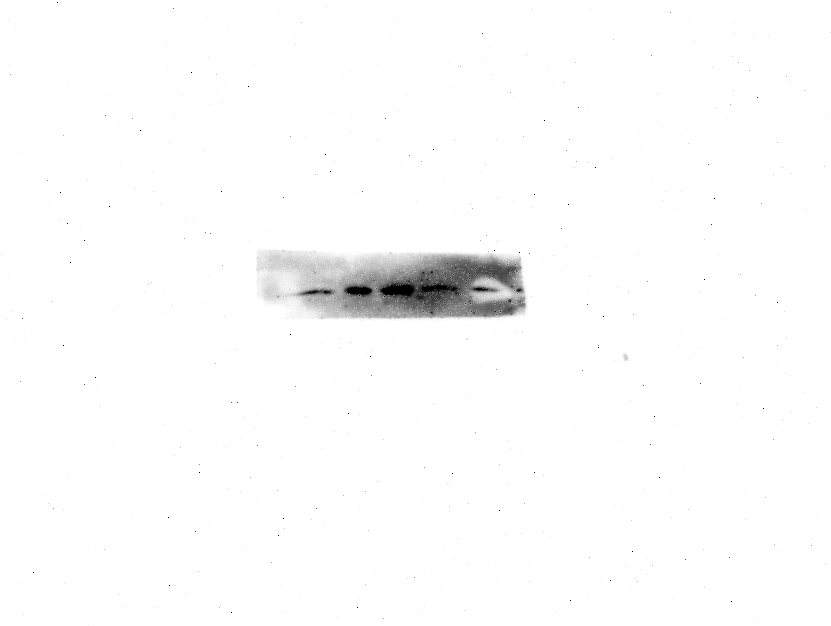

Supplement: Supplementary file 1 [file DataSheet_1.zip › Raw Date-1/Figure2/Figure2B/Western blot original images/p-IKB-α(39KD).png]

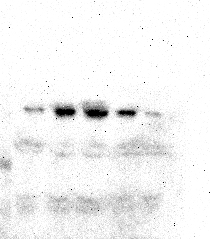

Supplement: Supplementary file 1 [file DataSheet_1.zip › Raw Date-1/Figure2/Figure2B/Western blot original images/p-p65(65KD).jpg]

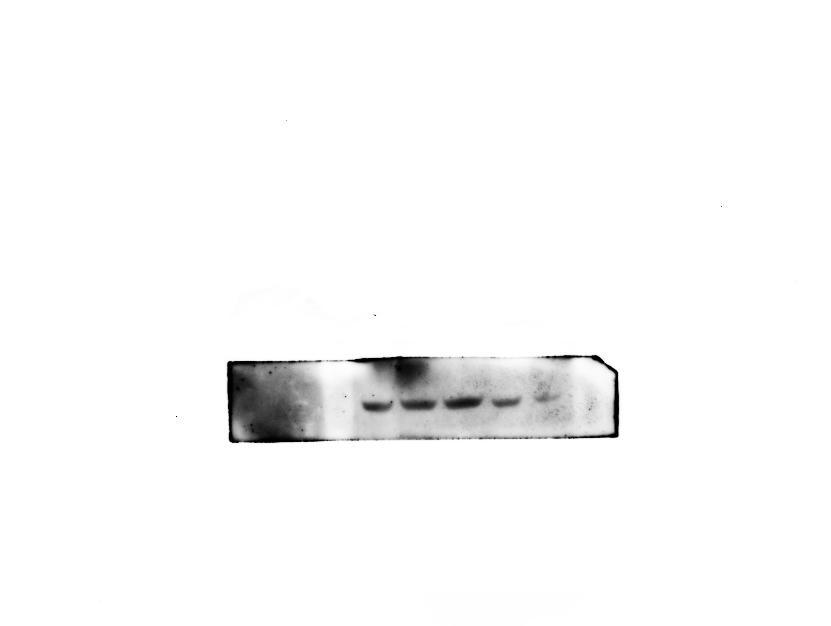

Supplement: Supplementary file 1 [file DataSheet_1.zip › Raw Date-1/Figure2/Figure2B/Western blot original images/p65(65KD).jpg]

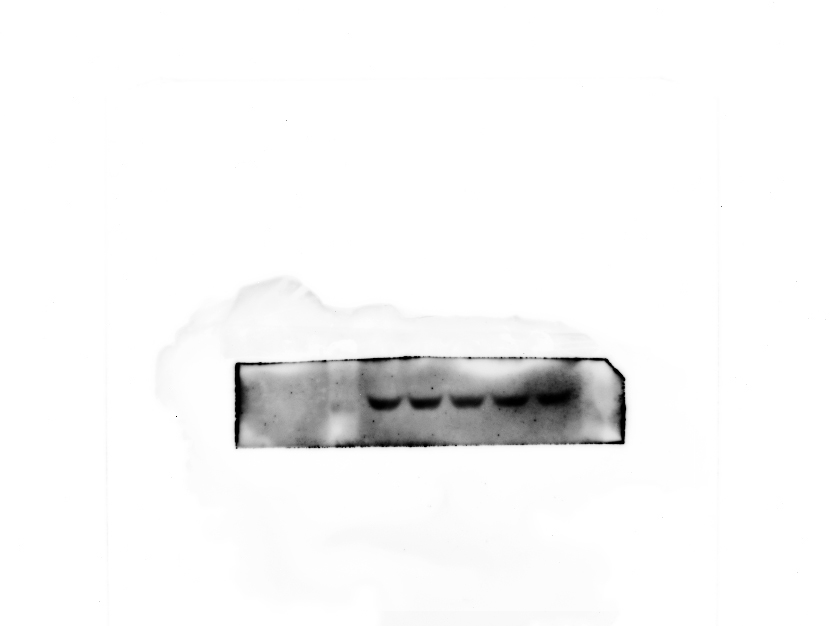

Supplement: Supplementary file 1 [file DataSheet_1.zip › Raw Date-1/Figure2/Figure2B/Western blot original images/β-actin(43KD).jpg]

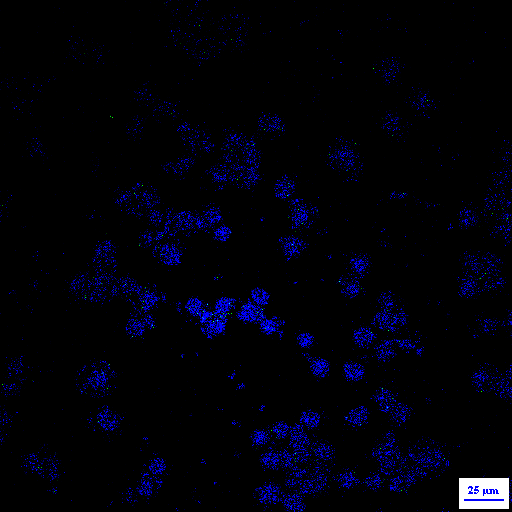

Supplement: Supplementary file 1 [file DataSheet_1.zip › Raw Date-1/Figure2/Figure2E/PBS Merge.tif]

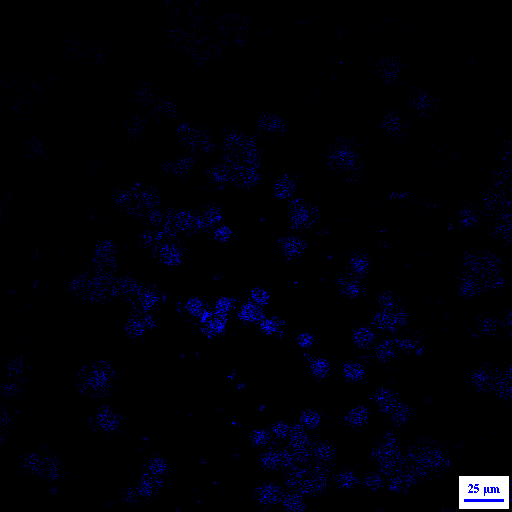

Supplement: Supplementary file 1 [file DataSheet_1.zip › Raw Date-1/Figure2/Figure2E/PBS Nucleus.tif]

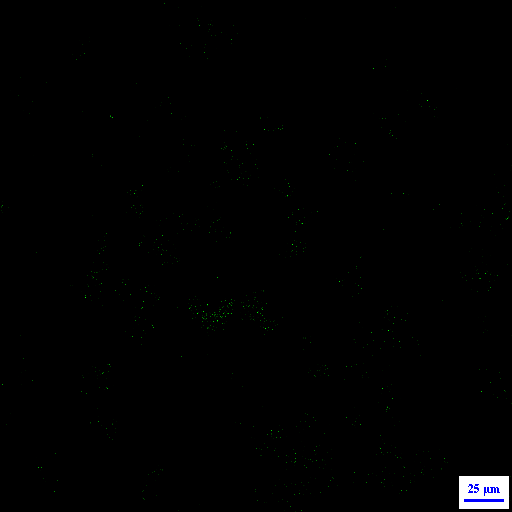

Supplement: Supplementary file 1 [file DataSheet_1.zip › Raw Date-1/Figure2/Figure2E/PBS p65.tif]

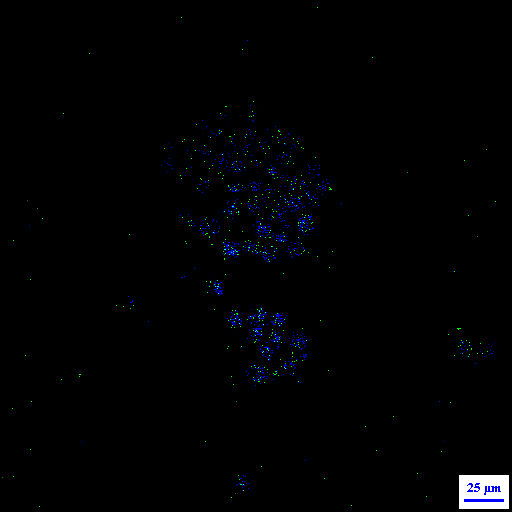

Supplement: Supplementary file 1 [file DataSheet_1.zip › Raw Date-1/Figure2/Figure2E/S2308 Merge.tif]

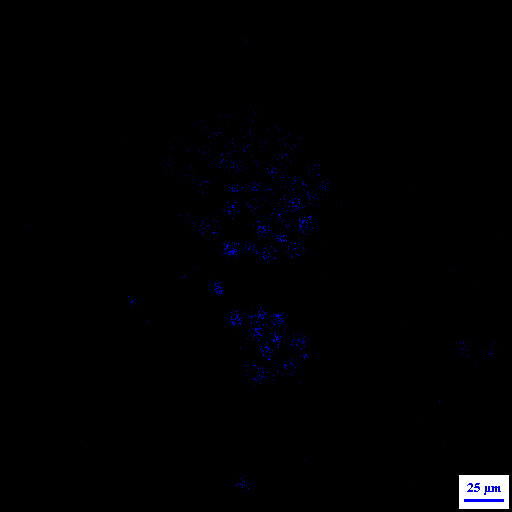

Supplement: Supplementary file 1 [file DataSheet_1.zip › Raw Date-1/Figure2/Figure2E/S2308 Nucleus.tif]

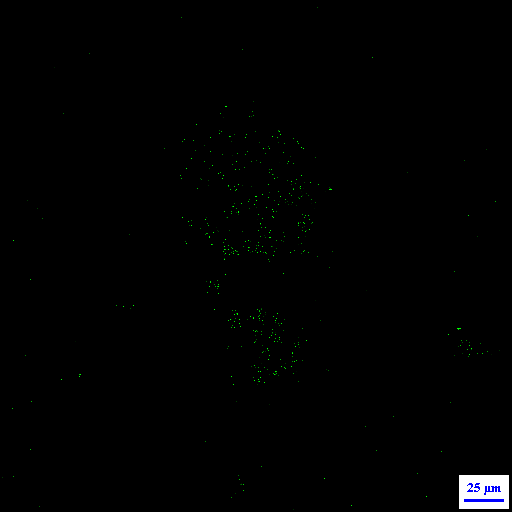

Supplement: Supplementary file 1 [file DataSheet_1.zip › Raw Date-1/Figure2/Figure2E/S2308 p65.tif]

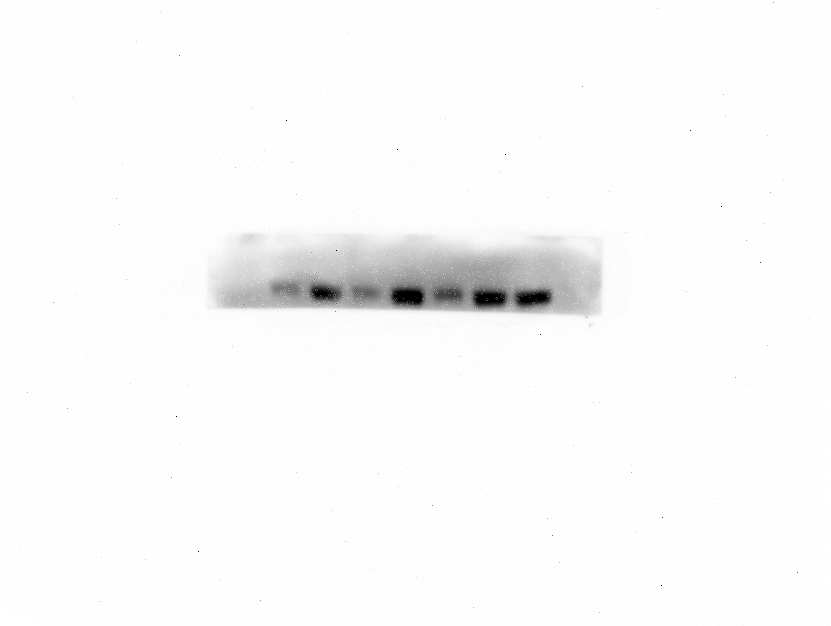

Supplement: Supplementary file 1 [file DataSheet_1.zip › Raw Date-1/Figure3/Figure 3B/Western blot original images/p-p65(65KD)12h.png]

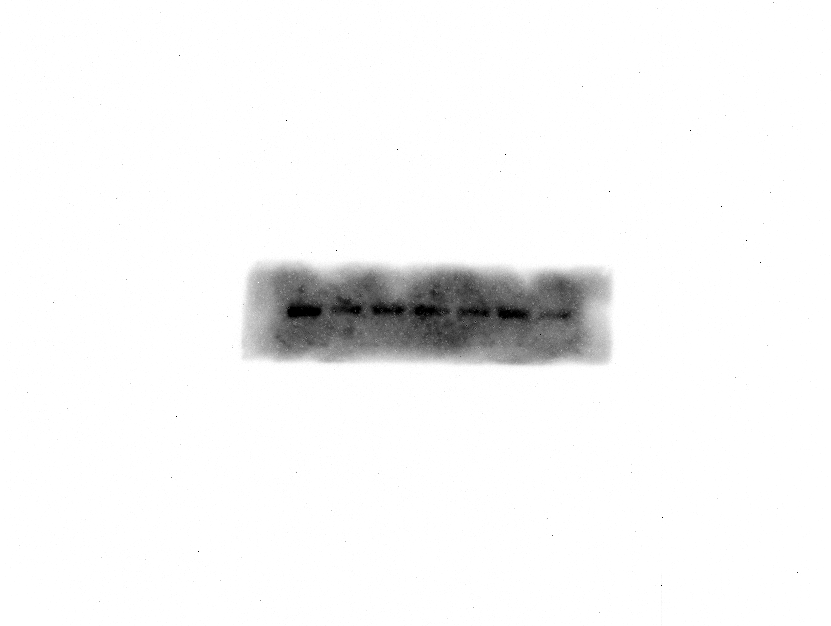

Supplement: Supplementary file 1 [file DataSheet_1.zip › Raw Date-1/Figure3/Figure 3B/Western blot original images/p-p65(65KD)48h.png]

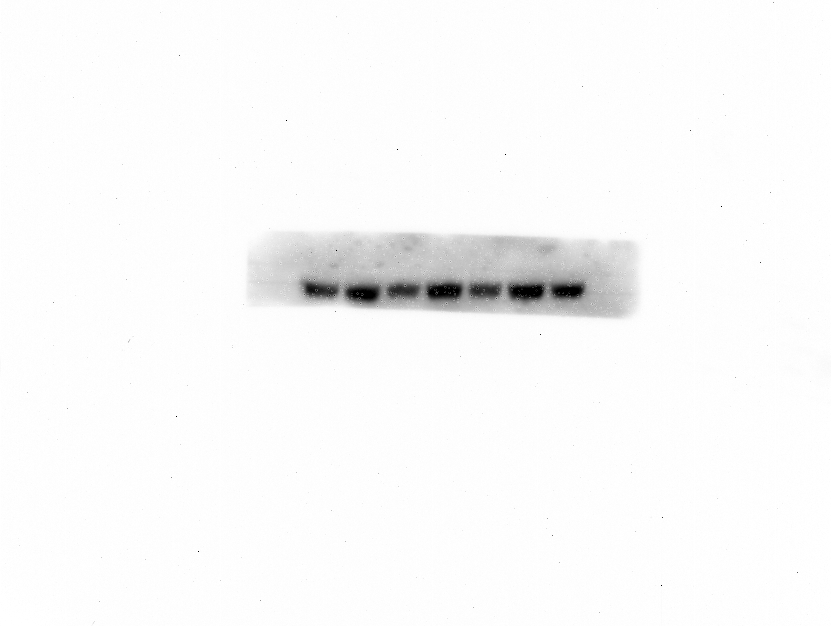

Supplement: Supplementary file 1 [file DataSheet_1.zip › Raw Date-1/Figure3/Figure 3B/Western blot original images/p65(65KD)12h.png]

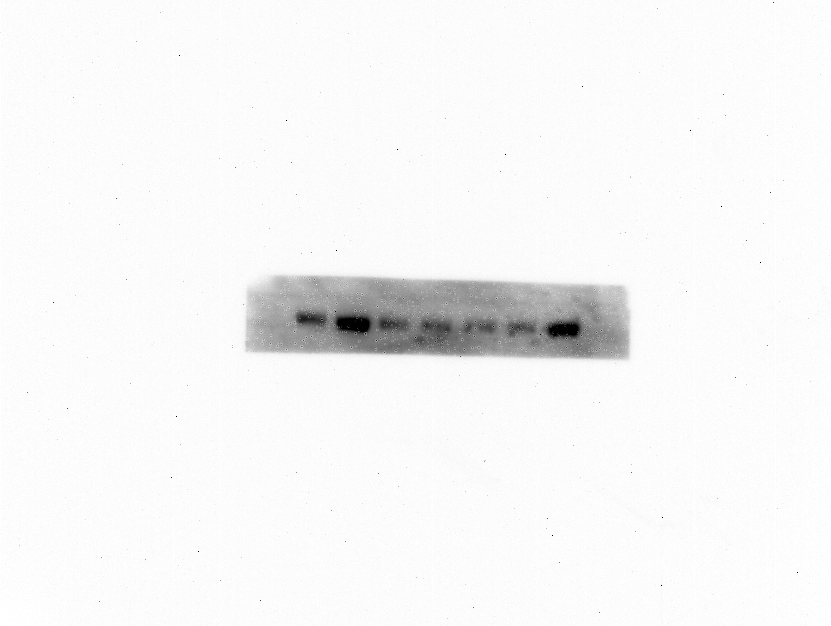

Supplement: Supplementary file 1 [file DataSheet_1.zip › Raw Date-1/Figure3/Figure 3B/Western blot original images/p65(65KD)48h.png]

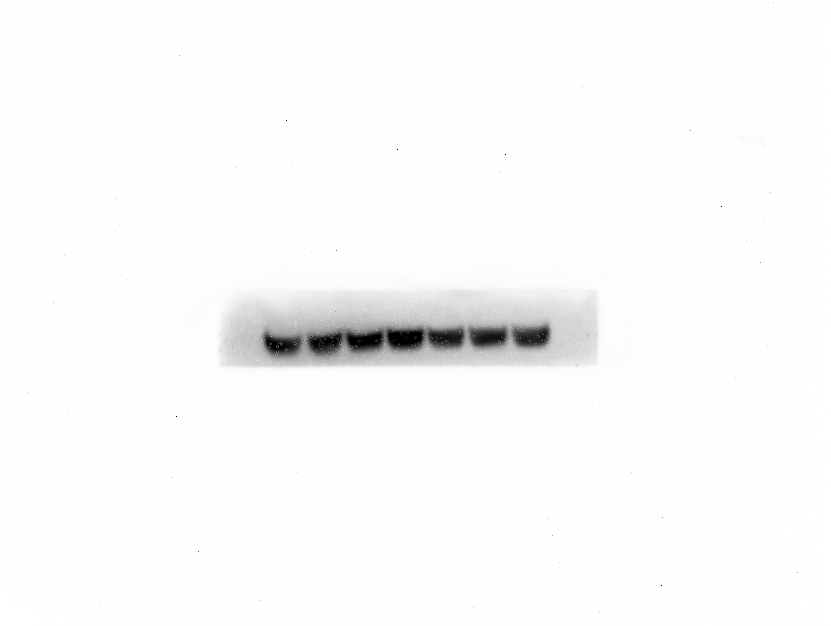

Supplement: Supplementary file 1 [file DataSheet_1.zip › Raw Date-1/Figure3/Figure 3B/Western blot original images/β-actin(43KD) 12h.png]

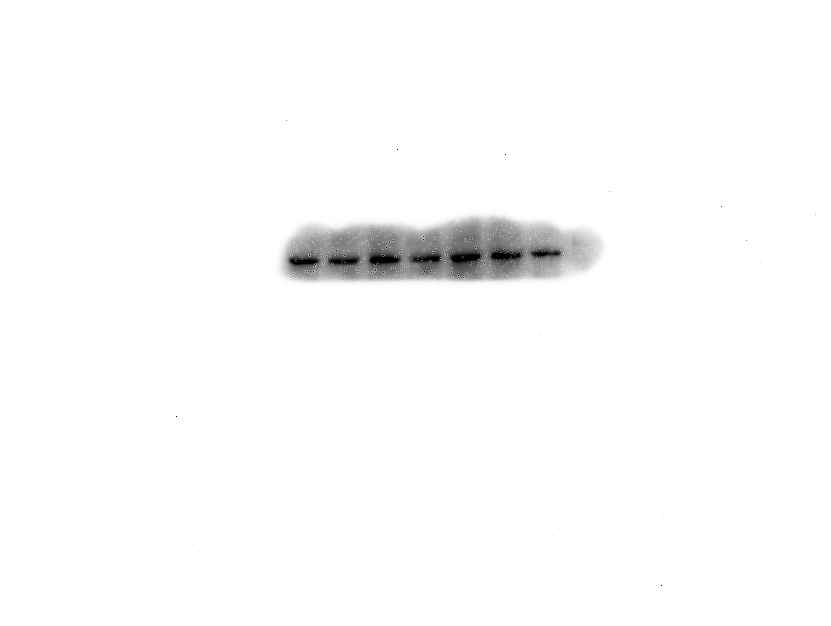

Supplement: Supplementary file 1 [file DataSheet_1.zip › Raw Date-1/Figure3/Figure 3B/Western blot original images/β-actin(43KD) 48h.png]

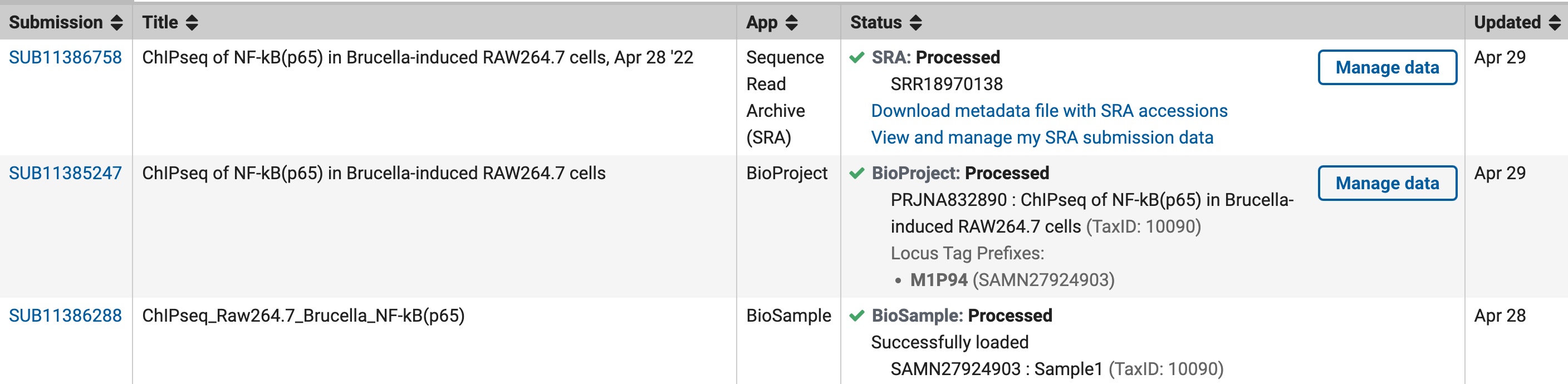

Supplement: Supplementary file 1 [file DataSheet_1.zip › Raw Date-1/Figure4/Figure4/CHIP-seq data upload to SRA database.jpg]

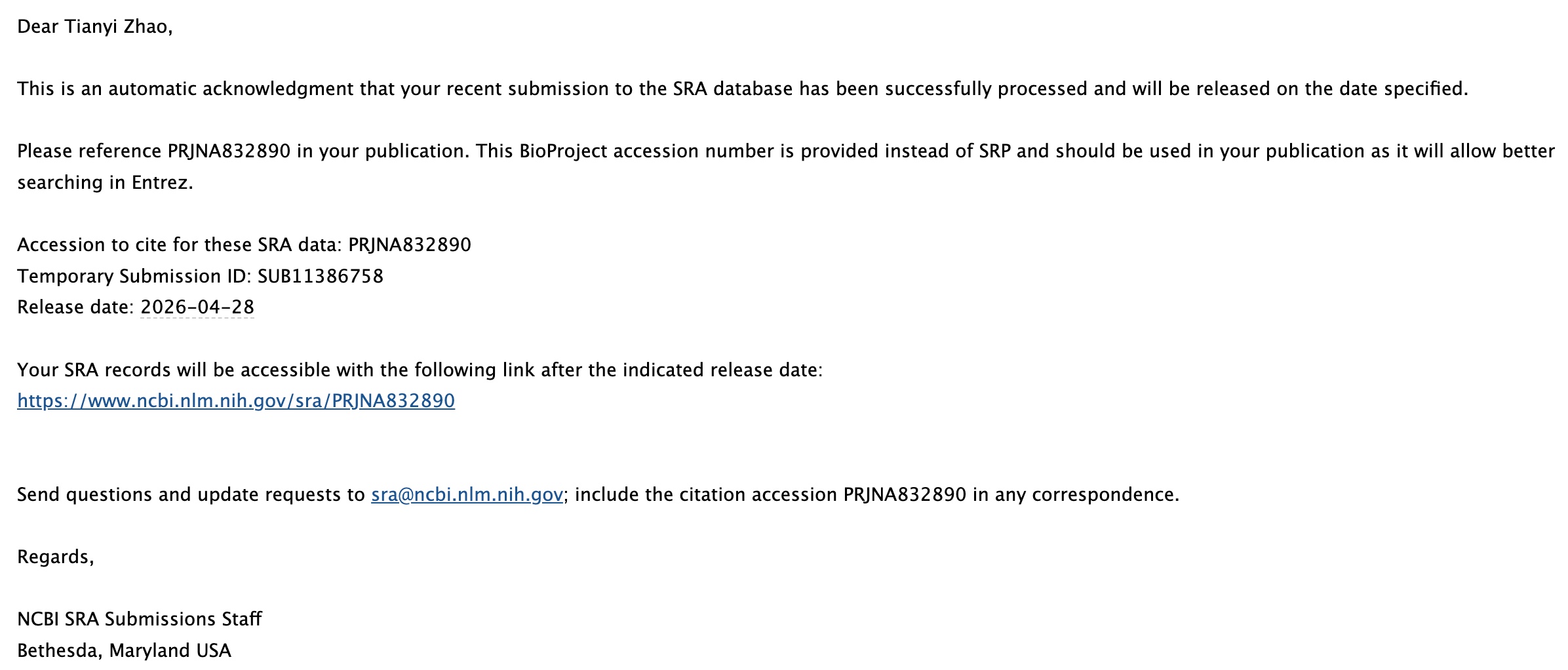

Supplement: Supplementary file 1 [file DataSheet_1.zip › Raw Date-1/Figure4/Figure4/Email Feedback CHIP-seq data uploaded to SRA database successfully.jpg]

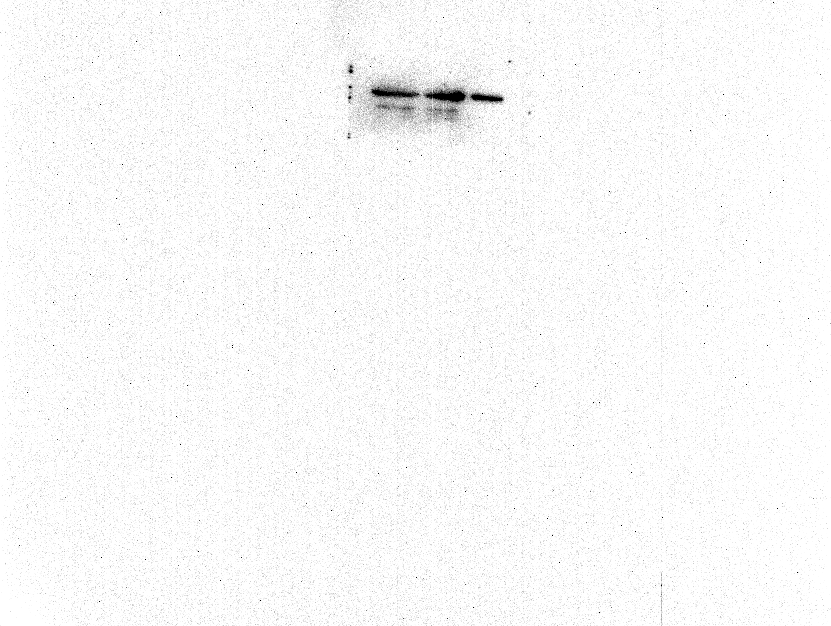

Supplement: Supplementary file 2 [file DataSheet_2.zip › Raw Date-2/Figure5/Figure5B/Western blot original images/GLS(71KD).png]

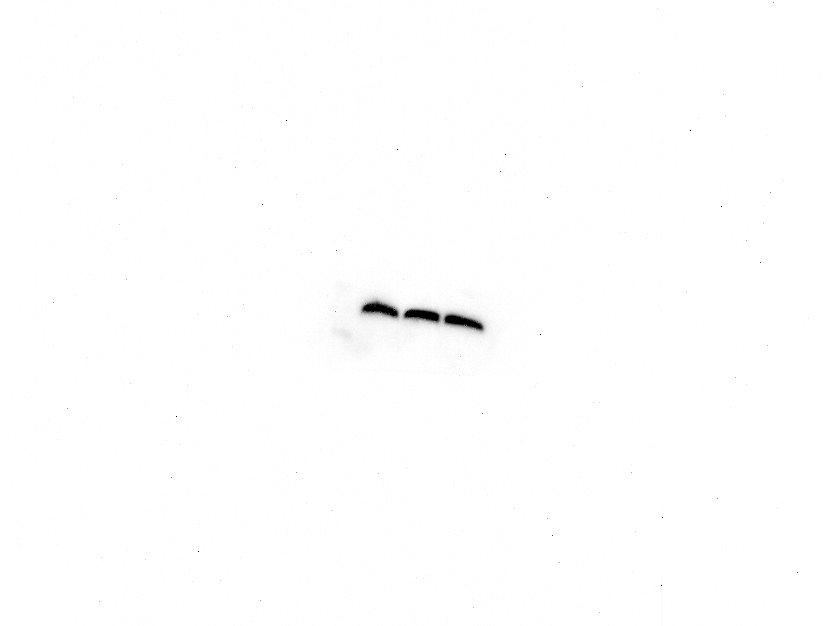

Supplement: Supplementary file 2 [file DataSheet_2.zip › Raw Date-2/Figure5/Figure5B/Western blot original images/β-actin(43KD).png]

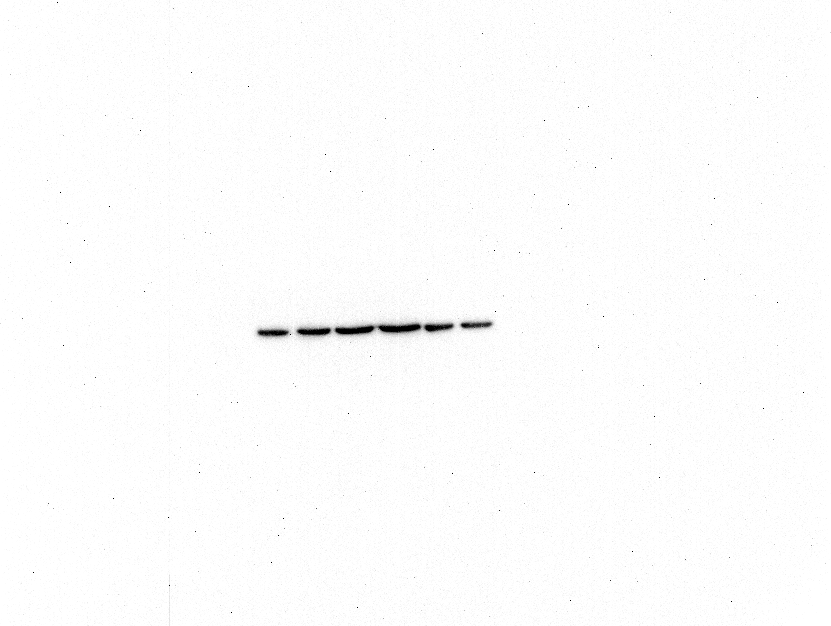

Supplement: Supplementary file 2 [file DataSheet_2.zip › Raw Date-2/Figure5/Figure5E/Western blot original images/GLS(71KD).png]

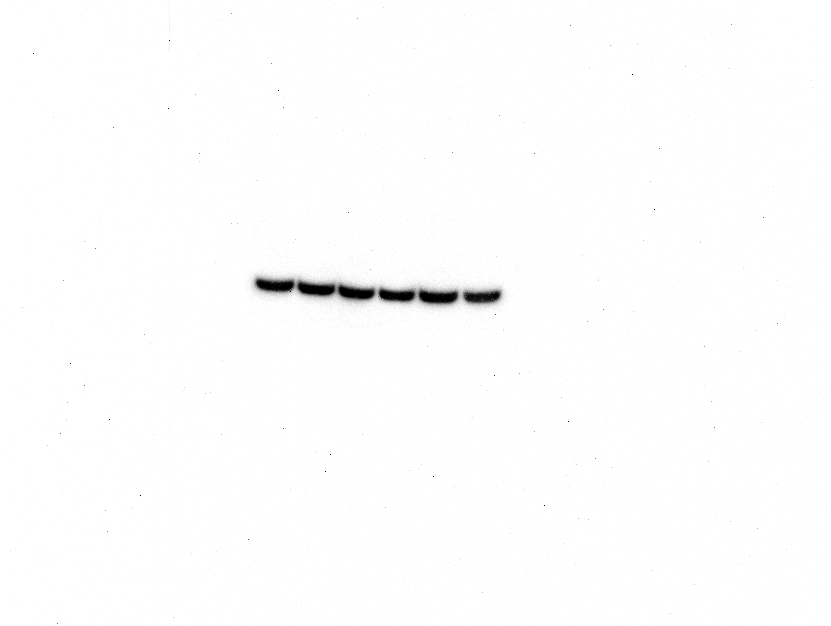

Supplement: Supplementary file 2 [file DataSheet_2.zip › Raw Date-2/Figure5/Figure5E/Western blot original images/β-actin(43KD).png]

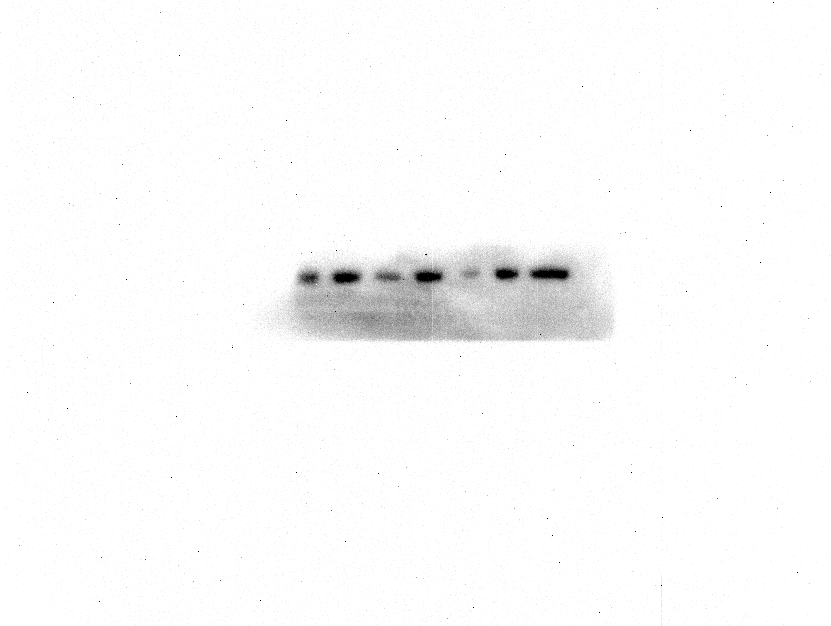

Supplement: Supplementary file 2 [file DataSheet_2.zip › Raw Date-2/Figure6/Figure 6B/Western blot original images/GLS (71KD) 12h.png]

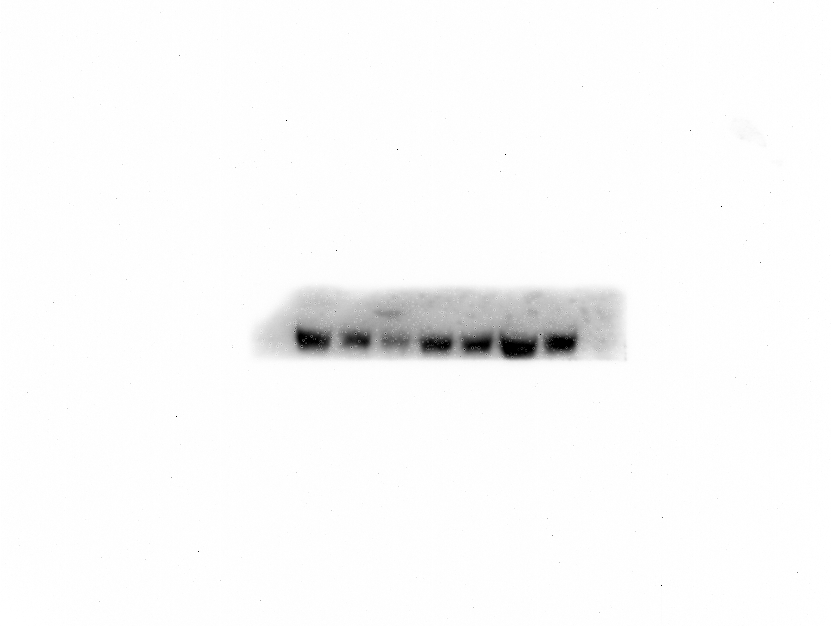

Supplement: Supplementary file 2 [file DataSheet_2.zip › Raw Date-2/Figure6/Figure 6B/Western blot original images/GLS (71KD) 48h.png]

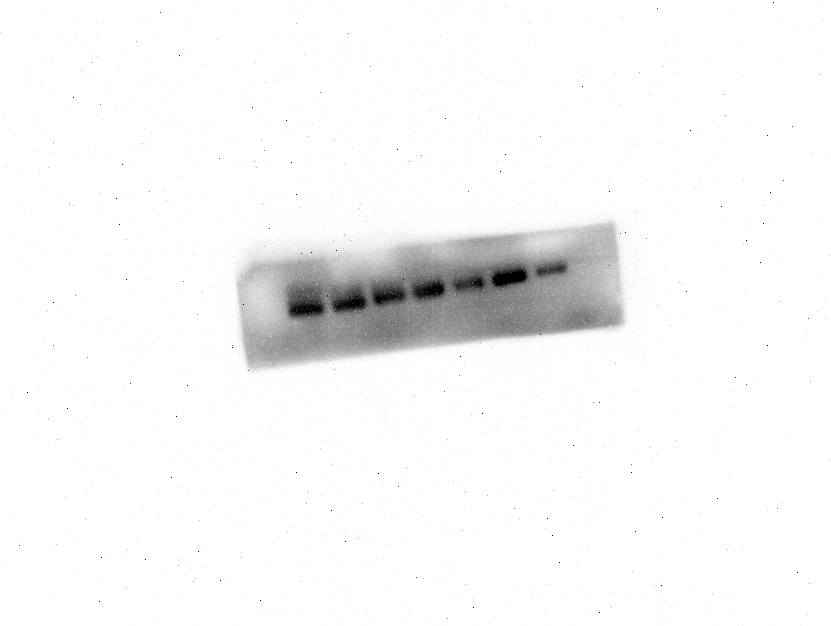

Supplement: Supplementary file 2 [file DataSheet_2.zip › Raw Date-2/Figure6/Figure 6B/Western blot original images/p-p65(65KD)12h.png]

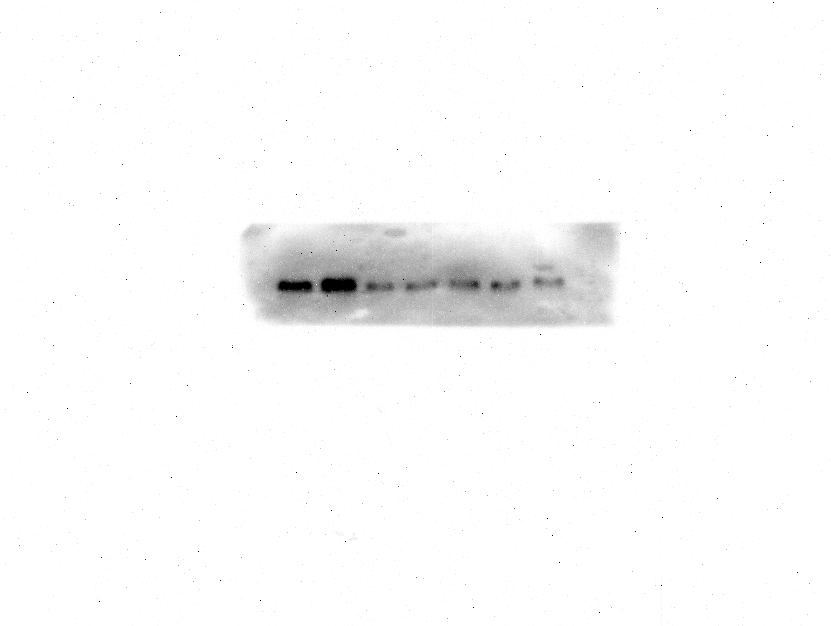

Supplement: Supplementary file 2 [file DataSheet_2.zip › Raw Date-2/Figure6/Figure 6B/Western blot original images/p-p65(65KD)48h.png]

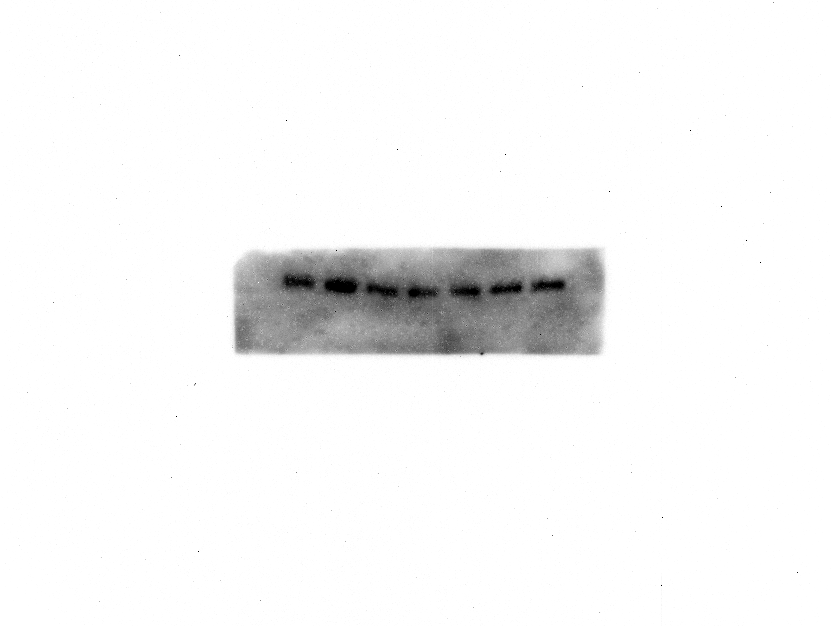

Supplement: Supplementary file 2 [file DataSheet_2.zip › Raw Date-2/Figure6/Figure 6B/Western blot original images/p65(65KD)12h.png]

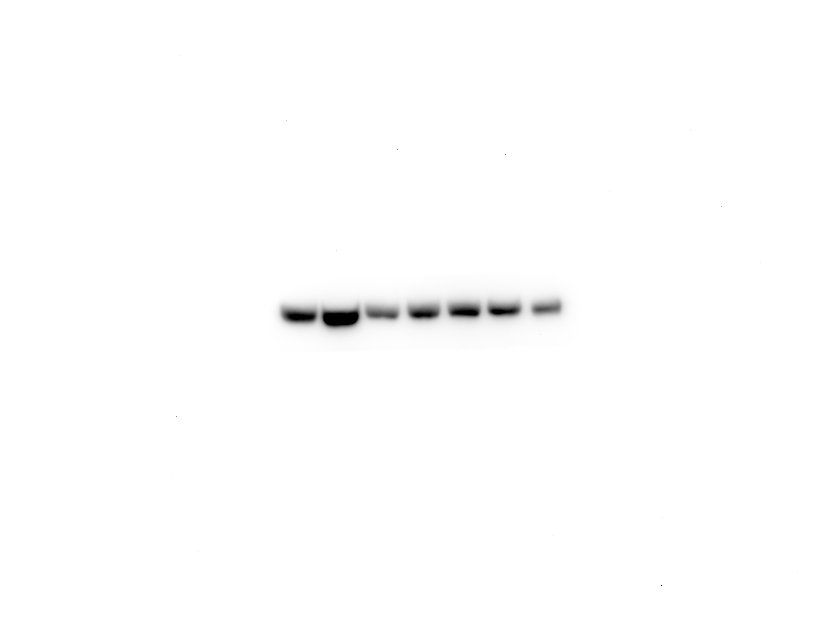

Supplement: Supplementary file 2 [file DataSheet_2.zip › Raw Date-2/Figure6/Figure 6B/Western blot original images/p65(65KD)48h.png]

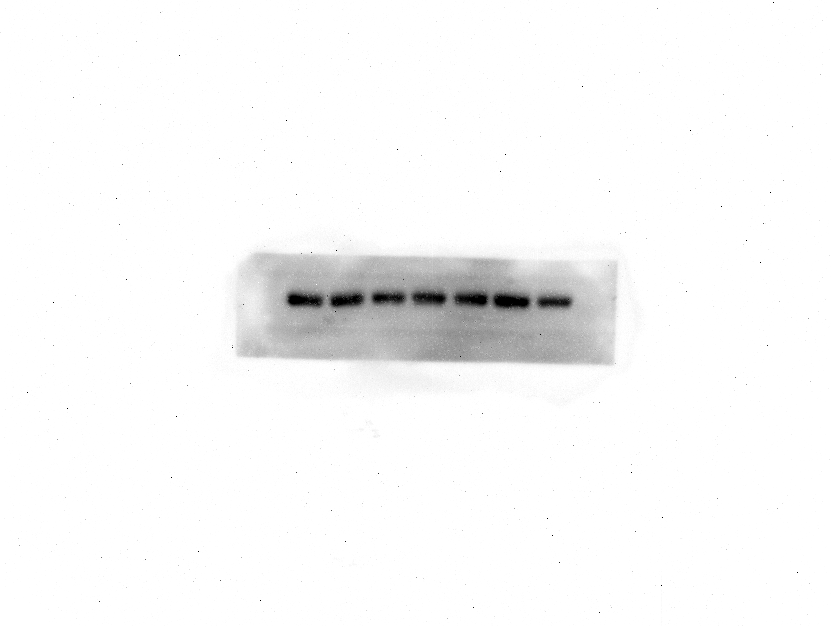

Supplement: Supplementary file 2 [file DataSheet_2.zip › Raw Date-2/Figure6/Figure 6B/Western blot original images/β-actin(43KD) 12h.png]

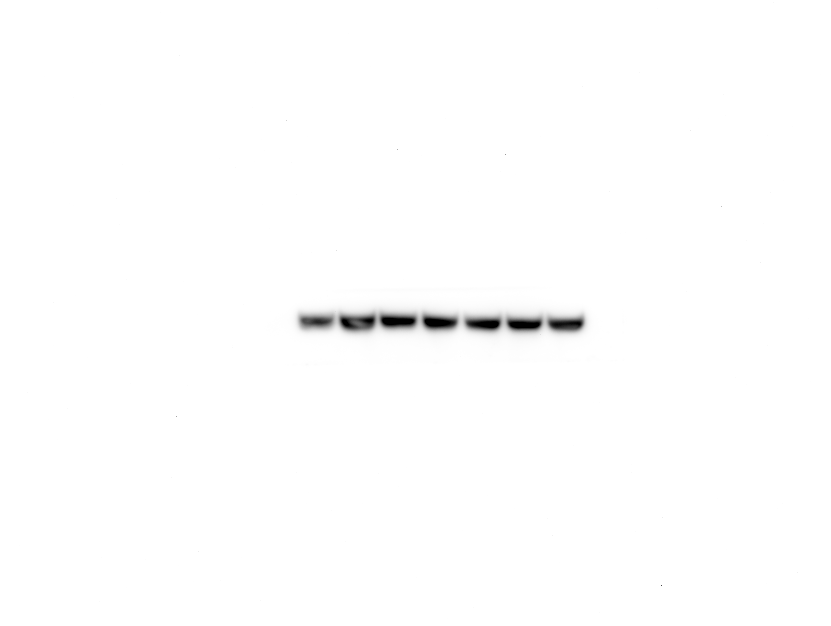

Supplement: Supplementary file 2 [file DataSheet_2.zip › Raw Date-2/Figure6/Figure 6B/Western blot original images/β-actin(43KD) 48h.png]

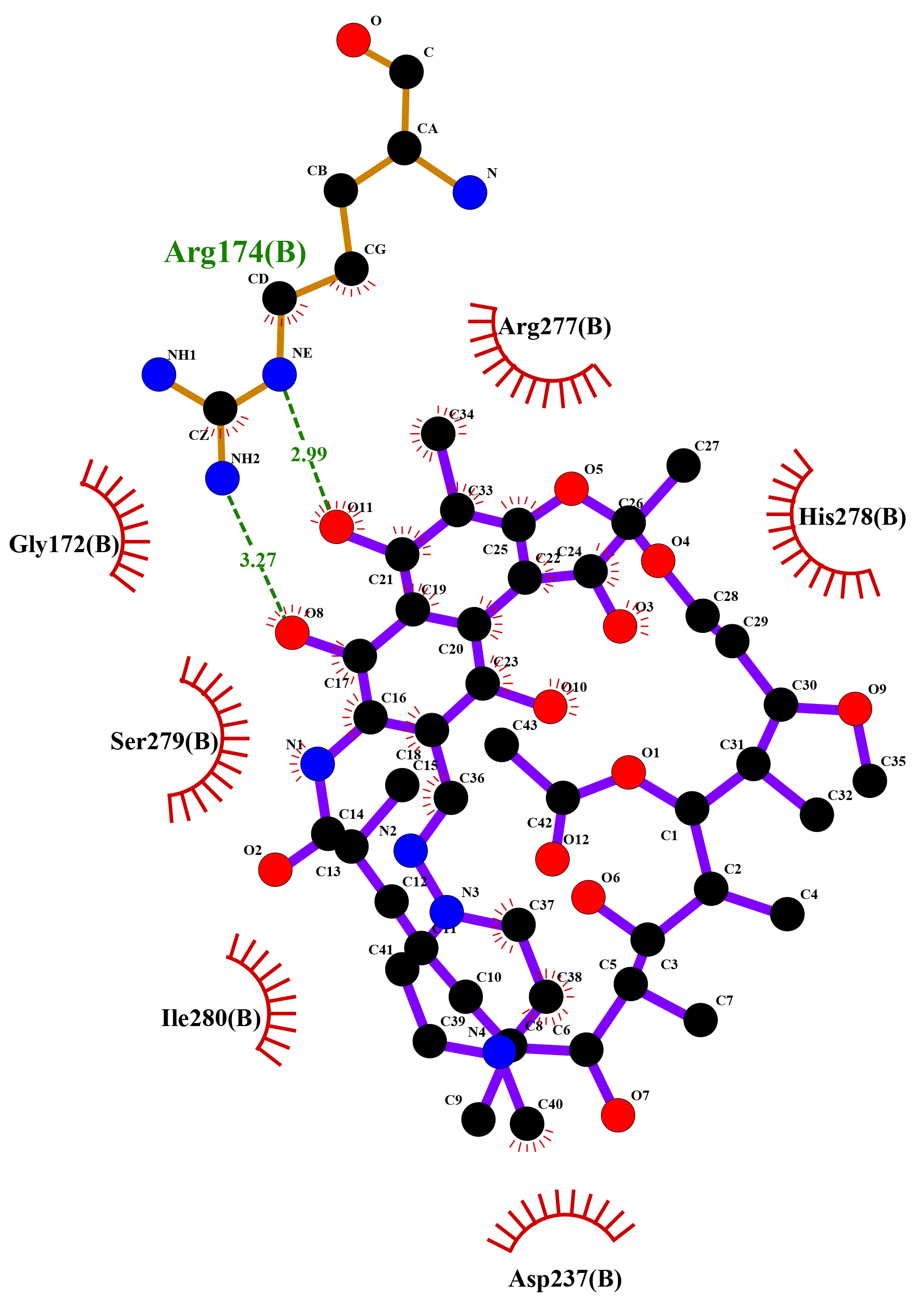

Supplement: Supplementary file 2 [file DataSheet_2.zip › Raw Date-2/Figure6/Figure 6I, J/figure6I.tif]

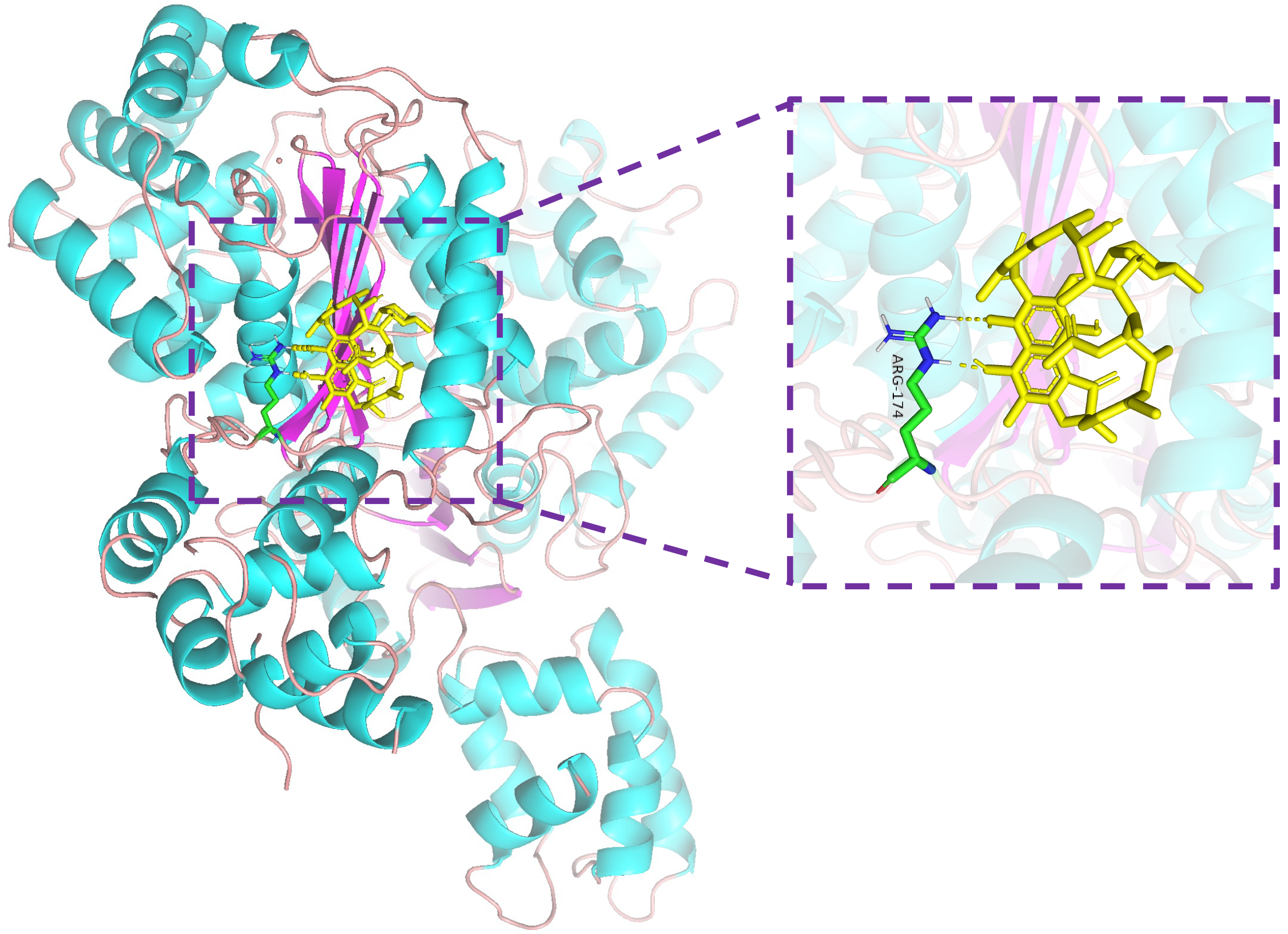

Supplement: Supplementary file 2 [file DataSheet_2.zip › Raw Date-2/Figure6/Figure 6I, J/figure6J.png]

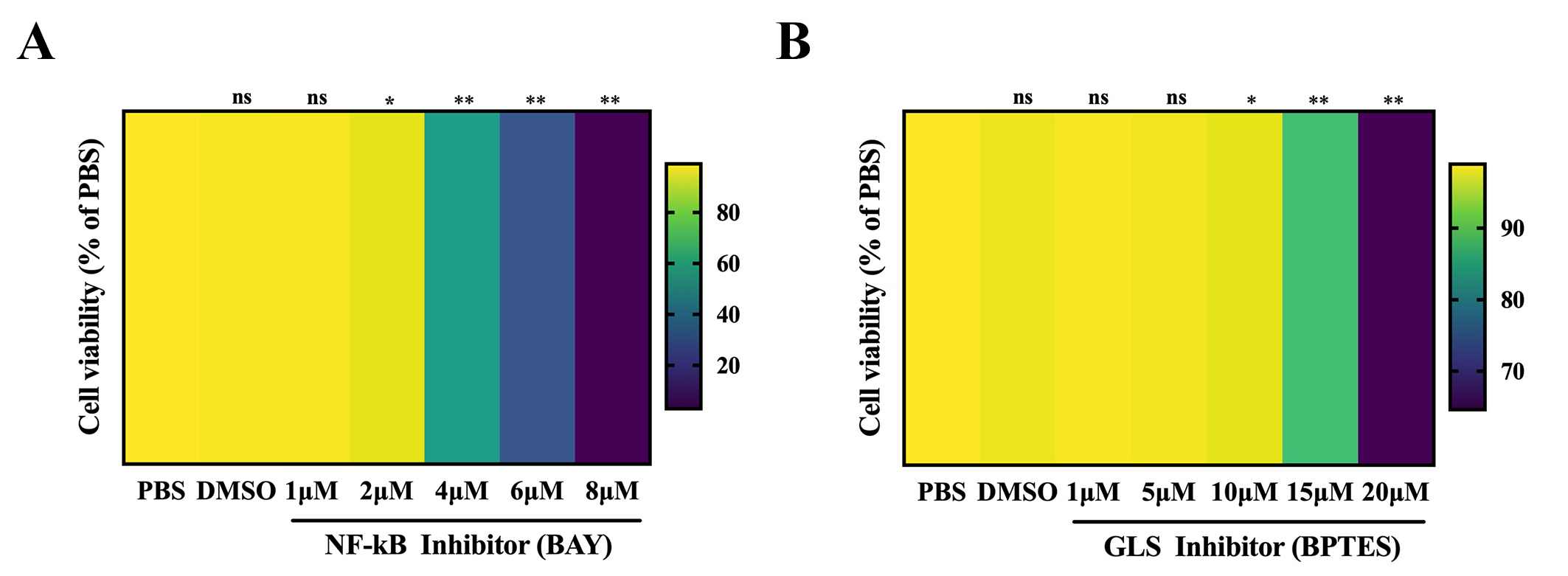

Supplement: Supplementary file 2 [file DataSheet_2.zip › Raw Date-2/Supplementary material/Figure S.jpg]
