# Supplementary material for: Brucella abortus modulates macrophage polarization and inflammatory response by targeting glutaminases through the NF-κB signaling pathway
Source: Front Immunol. 2023 May 31;14:1180837. doi: 10.3389/fimmu.2023.1180837 (PMC10266586; doi:10.3389/fimmu.2023.1180837)
Supplement: Supplementary file 2 [file DataSheet_2.zip › Raw Date-2/Certificate_of_editing-BYLRG_6_2_i1tf367.pdf]

editage

Editing Certificate

This document certifies that the manuscript listed below has been edited to ensure language and grammar accuracy and is error free in these aspects. The logical presentation of ideas and the structure of the paper were also checked during the editing process. The edit was performed by professional editors at Editage, a division of Cactus Communications. The sections titled references were not edited by Editage upon the author's request.

The author's core research ideas were not altered during the editing process. Editage guarantees the quality of editing with the assumption that our suggested changes have been accepted and the edited text has not been altered without the knowledge of our editors.

MANUSCRIPT TITLE

Brucella Modulates Macrophage Polarization and Inflammatory Response by Targeting Antagonistic Glutaminases Through the NF-κB Signaling Pathway

AUTHORS

Tianyi Zhao1†, Dexin Zhu1†, Zhihua Sun1, Liangbo, Liu1, Xingmei Deng1, Jia Guo1, Shuzhu Cao1, Yingjin Chai1, P. I. Baryshnikov1,2, Suleimenov Maratbek1,3, Zhen Wang1* and Hui Zhang1*

ISSUED ON

April 29, 2022

JOB CODE

BYLRG_6_2

THE EDITAGE Promise QUALITY GUARANTEE

Vikas Narang

Vikas Narang
Chief Operating Officer - Editage

editage

Editage, a brand of Cactus Communications, offers professional English language editing and publication support services to authors engaged in over 1300 areas of research. Through its community of experienced editors, which includes doctors, engineers, published scientists, and researchers with peer review experience, Editage has successfully helped authors get published in internationally reputed journals. Authors who work with Editage are guaranteed excellent language quality and timely delivery.

GLOBAL :
+1(833) 979-0061 | request@editage.com

CHINA :
400-120-3020 | fabiao@editage.cn

CACTUS

IMPACT SCIENCE impact.science

researcher.life

CACTUS LIFE SCIENCES lifesciences.cactusglobal.com
