# Supplementary material for: Brucella abortus modulates macrophage polarization and inflammatory response by targeting glutaminases through the NF-κB signaling pathway
Source: Front Immunol. 2023 May 31;14:1180837. doi: 10.3389/fimmu.2023.1180837 (PMC10266586; doi:10.3389/fimmu.2023.1180837)
Supplement: Supplementary file 2 [file DataSheet_2.zip › Raw Date-2/Supplementary material/Supplementary Table 1.docx]

**Supplementary Table 1**

**A**

Primers used in qRT-PCR

| Gene name | Primer | |
| --- | --- | --- |
| *NF-kB p65* | F: AGGCTTCTGGGCCTTATGTG | R: TGCTTCTCTCGCCAGGAATAC |
| *TNF-α* | F: GACGTGGAACTGGCAGAAGAG | R: TTGGTGGTTTGTGAGTGTGAG |
| *NOS2* | F: CAGCTGGGCTGTACAAACCTT | R: CATTGGAAGTGAAGCGTTTCG |
| *IL-1β* | F: GCTTCAGGCAGGCAGTAT | R: ACAAACCGCTTTTCCATCT |
| *TGF-β* | F: CTCCCGTGGCTTCTAGTGC | R: GCCTTAGTTTGGACAGGATCTG |
| *ARG1* | F: CATATCTGCCAAAGACATCGTG | R: GACATCAAAGCTCAGGTGAATC |
| *IL-10* | F: TTCTTTCAAACAAAGGACCAGC | R: GCAACCCAAGTAACCCTTAAAG |
| *Gls* | F: CTACAGGATTGCGAACATCTGAT | R: ACACCATCTGACGTTGTCTGA |
| *GAPDH* | F: AGGTCGGTGTGAACGGATTTG | R: TGTAGACCATGTAGTTGAGGTCA |

**B**

>NC_000067.7:c52225042-52224742 Mus musculus strain C57BL/6J chromosome 1, GRCm39

TAAACTTCAAACTATTTTGTTAATTTTTGGCACTTCCTATATATAATTCTAGTAATGCTTGAATGTACACTTAAATATGAAGTAGGATTAAGTCAGCTGCTGTGTTTAAAGAATGCTGTTAAGAACAAGCATTCAAAACTGTATAGGAAGGTATTAGCTTAAGAGTAGGTAAGATACCGTGACTGTATCTGCAGACAAGAAGAGGAAAGAAAAGCTTTGCCAGTTTGTGGATTTATCTTAATTCCCTTCAGTATATTCAATCTCTTTTCAAATAAAGCTCTTTGAGAAGTACCCAGTATTG
